# Supplementary material for: Household storage, surplus and supra-household storage in prehistoric and protohistoric societies of the Western Mediterranean
Source: PLoS One. 2020 Sep 14;15(9):e0238237. doi: 10.1371/journal.pone.0238237 (PMC7489512; doi:10.1371/journal.pone.0238237)
Supplement: S1 Text — (DOCX) [file pone.0238237.s001.docx]

**S1. Definitions**

- *Dispersed settlement:* a model of settlement with a high scattering of dwellings, no complexity with regard to space, and an expansive use of the space. It is defined as having a "loose" organisation in which each productive unit possesses a certain degree of autonomy (López 2000: 172; Francès 2005: 62).

- *Farming system*: we rely upon previous systematization by Bogaard to define two main agricultural models: intensive and extensive farming (Bogaard 2005: 179-180). *Intensive farming* refers to permanent fields/plots with a high investment of labour per unit of land applying techniques including crop rotation or fertilisation with animal manure. Due to manpower, this results in a greater yield from the fields, a production that generally covers the needs of a domestic unit. It is a small-scale operation that depends on the labour capacity of the domestic unit, and a relatively small number of animals that remain near the settlement. The crop yields forage for the livestock that, in turn, supplies manure for the fields and through grazing regulates the growth of crops. *Extensive farmin*g, by contrast, involves the cultivation of larger fields with a lower input of labour per unit of land, usually connected to the use of draught animals. This type is linked to a type of livestock that is managed in large flocks that graze over great surfaces and usually is intentionally detached from the agricultural system, in opposition to intensive farming models.

- *Household / Domestic unit*: is a unit of economic and social cooperation. Group of people that does not necessarily live under a single roof (dwelling unit), though it often does. They cooperate and share economically on a daily basis a number of activities, including one or more of the following: production, consumption, pooling of resources, distribution, transmission, coresidence, reproduction and shared ownership (Wilk & Rathje 1982: 620; 621; Ashmore & Wilk 1988: 6). A nuclear type is a group of 5-7 economically autonomous individuals; and an extensive type is a group with a number surpassing that of a nuclear unit (Nimkoff & Middleton 1960; Kramer 1982: 121; Belarte 2013: 87).

- *Nucleated settlement*: a model of settlement that is concentrated and formed by veritable agglomerations comprising nuclei of stone or earth dwellings. This settlement type denotes a lasting and permanent occupation (Delibes de Castro & Romero 1992: 243).

- *Silo fields*: Group of underground storage structures in an area separated from other features of the settlement. These types of clusters appear since the Early Iron Age. According to the available agricultural data, they respond mainly to the storage of surplus products since their volume exceeds the needs of immediate consumption. This definition also applies to groups of silos prior to this period that are found in similar large clusters or concentrations (Sanmartí 2015).

- *Productivity*: relation between the amount of products and the elements used to obtain them in a certain timeframe. Extensive cultivation/agricultural system using the plough or the technique of slash and burn. Cultivation of fields larger than those of the previous system (intensive), which generates less productivity per unit of land.
