# Supplementary material for: Household storage, surplus and supra-household storage in prehistoric and protohistoric societies of the Western Mediterranean
Source: PLoS One. 2020 Sep 14;15(9):e0238237. doi: 10.1371/journal.pone.0238237 (PMC7489512; doi:10.1371/journal.pone.0238237)
Supplement: S1 Table — (DOCX) [file pone.0238237.s002.docx]

**S1 Table. Sites list and type of settlement.**

| SETTLEMENT NAME | TYPE OF SETTLEMENT |
| --- | --- |
| Minferri | Open air settlement |
| Cantorella | Open air settlement |
| Molins de la Vila | Open air settlement |
| El Cavet | Open air settlement |
| La Masieta de Queralt | Isolated silo-pit |
| L’Era del Castell | Fortified settlement |
| Aeroport de Reus | Open air settlement |
| Can Sadurní | Cave/rock shelter |
| Camí de Cal Piques | Open air settlement |
| Pou Nou-2 | Open air settlement |
| El Pujolet de Moja | Open air settlement |
| Estinclells | Fortified settlement |
| Els Pujols/Serra de la Pairal | Open air settlement |
| Camí de Santa Maria dels Horts | Open air settlement |
| Sitges Carrer Elisenda | Silo field |
| Camp Cinzano | Open air settlement |
| La Girada | Open air settlement |
| Els Cirerers | Open air settlement |
| Mas d’en Boixos-1 | Open air settlement |
| Vinya del Ticó | Open air settlement/silo field |
| Feixa Llarga | Open air settlement |
| Pla de la Bruguera | Open air settlement |
| Els Mallols | Open air settlement |
| Can Piteu I | Open air settlement |
| Hort d’en Grimau | Open air settlement |
| Can Filuà | Open air settlement |
| Pinetons I | Open air settlement |
| Pinetons II | Open air settlement |
| Antic Vapor Gorina | Open air settlement |
| Can Gambús 3 | Open air settlement |
| Institut de Batxillerat Antoni Pous/Institut de Manlleu | Open air settlement |
| Sant Joan Nord | Open air settlement/silo field |
| Escola bressol de la Florida Nord | Open air settlement |
| Can Pona | Silo field |
| Bòbila Madurell - Can Feu | Open air settlement |
| Pla de Tabac I i II | Open air settlement |
| Llirians del Mas, Les Torres i Sector Rec | Open air settlement |
| Vinya del Corb | Open air settlement |
| El Collet | Open air settlement |
| Espina C | Open air settlement |
| Els Vilars | Fortified settlement |
| Serrat dels Espinyers | Open air settlement/silo field |
| Barranc del Prat | Open air settlement |
| Rosella | Open air settlement/silo field |
| Mas d’en Gual | Open air settlement/silo field |
| Missatges | Silo field |
| Horts de Can Torras | Open air settlement |
| Can Fatjó | Open air settlement/silo field |
| Parc del Castell de Rubí | Silo field |
| Can Gambús 1 | Open air settlement |
| Can Gambús 2 | Open air settlement |
| Cinc Ponts | Open air settlement |
| Mas Pujó | Open air settlement |
| La Serreta | Open air settlement |
| Santa Digna III | Open air settlement |
| Can Xercavins | Open air settlement |
| Sitja del Camí del Mig | Isolated silo-pit |
| Planell de Sanaüja | Open air settlement |
| Quatre Pilans I | Open air settlement |
| Sindreu 1 | Open air settlement |
| Cova Colomera | Cave/rock shelter |
| Sitges de la UAB | Open air settlement |
| Can Badell/Can Vedell/CEIP el Turó | Silo field |
| Can Ballarà | Isolated silo-pit |
| Montjuïc | Silo field |
| Carrer de la Rectoria, 16 | Silo field |
| Facultat de Medicina de la UAB | Open air settlement |
| Cal Jardiner I | Open air settlement |
| Sitges | Open air settlement |
| Ca l’Almell de la Muntanya | Isolated silo-pit |
| Santa Creu d’Olorda | Isolated silo-pit |
| Turó de la Rovira | Fortified settlement |
| Can Miralles - Can Modolell (Oppidum Burriac) | Silo field |
| Cadira del Bisbe | Fortified settlement |
| Can Tintorer | Silo field |
| Penya del Moro | Open air settlement |
| Torrebonica | Open air settlement |
| Mas Vilalba | Open air settlement/silo field |
| Mas Castellar | Fortified settlement/silo field |
| Camp de l’Ylla | Isolated silo-pit |
| Saus II/Camp d’en Dalmau | Silo field |
| Vial del port | Silo field |
| Banys de la Mercè | Open air settlement |
| Poblat ibèric de Castell | Fortified settlement/silo field |
| Camp d’en Gou/Gorg d’en Batlle | Silo field |
| Camp d’en Pitu Porusia | Silo field |
| Camp Gran | Silo field |
| Camp de l’Abadia | Silo field |
| Camp de Mas Figueres | Open air settlement |
| Mas Boscosa | Isolated silo-pit |
| Turó de la Bateria o Puig d’en Roca III - Vials | Open air settlement |
| Mas Xirgu Sud | Open air settlement |
| Mas Vedruna | Isolated silo-pit |
| Camp del Pla de Sant Esteve | Silo field |
| Turó de la Bateria / Hotel Ibis | Open air settlement |
| Torre Vedruna | Silo field |
| Can Serra | Silo field |
| Torre Vedruna - Sector Transports | Silo field |
| Olivet d’en Pujol | Open air settlement |
| Sitges de la carretera d’Aiguaviva | Silo field |
| Parc Residencial Vilacolum | Silo field |
| Prolongació del carrer de la Pau | Silo field |
| Empúries | Silo field |
| El Vilar | Fortified settlement |
| Turó de la Font de la Canya | Fortified settlement/silo field |
| C. de les Beates, 2-5 | Open air settlement |
| Carrers Reina Amàlia 31-33, Lleialtat 1-9 i Carretes 46 i 58 | Open air settlement |
| C. Riereta, 37-37 bis / C. Sant Pau, 84 | Open air settlement |
| Mercat de Santa Caterina | Open air settlement |
| Sitja de la Rambla Onze de Setembre | Isolated silo-pit |
| Can Soldevila | Open air settlement |
| Can Roqueta - DIASA | Open air settlement |
| Can Roqueta | Open air settlement |
| Estació de bombeig EDAR Sabadell-riu Ripoll (Roqueta) | Open air settlement |
| Les Guàrdies | Open air settlement/silo field |
| Sant Esteve d’Olius | Fortified settlement/silo field |
| Puig de Sant Andreu | Fortified settlement |
| Pla de les Sitges del Camaró | Silo field |
| Can Roqueta II (est) | Open air settlement |
| Hospital de la Santa Creu i Sant Pau | Silo field |
| LAV estació de la Sagrera | Open air settlement |
| Can Roqueta/Can Revella | Open air settlement |
| Pla del Serrador | Open air settlement |
| Carrer Ca n’Alzina - Carrer del Mas Carbó (Can Roqueta) | Open air settlement |
| Carrer Can Camps/Avinguda Can Bordoll (Can Roqueta) | Open air settlement |
| Far de Sant Sebastià | Silo field |
| Sant Sebastià de la Guarda | Open air settlement |
| Sitja de ca l’Angusto | Isolated silo-pit |
| Camps de Can Colomer | Open air settlement/silo field |
| Camp del Colomer | Open air settlement |
| Plaça de Sant Andreu | Isolated silo-pit |
| Camp de l’Arrencada | Silo field |
| Puig del Castell | Fortified settlement |
| Turó de Ca n’Oliver | Fortified settlement/silo field |
| Bòbila Madurell - Mas Duran | Open air settlement |
| La Pleta | Open air settlement |
| Bosc del Congost | Silo field |
| Castell de Rubí | Open air settlement |
| Can Roqueta/Torre-Romeu | Open air settlement |
| Can Fatjó dels Aurons | Open air settlement |
| Masia Can Roqueta - Edifici Annex | Open air settlement |
| Can Sant Joan | Open air settlement |
| Sant Miquel de Sorba | Open air settlement/silo field |
| Cova de la Pólvora | Cave/rock shelter |
| Plaça de la Constitució | Open air settlement |
| Casc Antic | Silo field |
| C/Alou, núm. 43- 47 i C/ Mossèn Francesc Albertí, núm. 1- 3 | Silo field |
| C. Hostal del Pi, polígon Barcelonès | Silo field |
| Camp de les Lloses | Silo field |
| Aiguacuit | Open air settlement |
| La Pedrera | Fortified settlement |
| Les Goges | Open air settlement |
| El Molàs | Open air settlement |
| Mas Boscà | Fortified settlement |
| Camí de Can Segarra-Can Bartomeu (Burriac) (=Can Bartomeu) | Silo field |
| Oppidum de Burriac (poblat + sitges) | Fortified settlement/silo field |
| Poblat ibèric de Castellvell | Fortified settlement/silo field |
| Camp del Rector | Open air settlement |
| Sant Llorenç de Boada-el Graell | Silo field |
| Bosc del Quer | Open air settlement |
| Can Bonells | Open air settlement |
| Font d’Abril/Font de Bril | Open air settlement |
| Turó del Vent | Fortified settlement/silo field |
| Castellvell | Silo field |
| Coll Blanc | Open air settlement |
| Puig Castellar | Fortified settlement/silo field |
| Guissona (fosses del camp 1) | Silo field |
| Font del Ros | Open air settlement |
| Mas Castellar | Fortified settlement/silo field |
| Sector industrial el rentador. Sector sud | Silo field |
| Vil·la romana de Sant Amanç | Open air settlement |
| El Castellot | Fortified settlement |
