# Supplementary material for: Household storage, surplus and supra-household storage in prehistoric and protohistoric societies of the Western Mediterranean
Source: PLoS One. 2020 Sep 14;15(9):e0238237. doi: 10.1371/journal.pone.0238237 (PMC7489512; doi:10.1371/journal.pone.0238237)
Supplement: S2 Table — (DOCX) [file pone.0238237.s003.docx]

**S2 Table. List of Ø/D index ranges and the total number of pits for each range and chronological period.**

| **RANGE** | **Early Neolithic** | **Middle Neolithic** | **Late Neolithic – Chalcolithic** | **Early Bronze Age** | **Late Bronze Age** | **Early Iron Age** | **Early Iberian** | **Middle Iberian** | **Late Iberian** |
| --- | --- | --- | --- | --- | --- | --- | --- | --- | --- |
| **< 0,5** | 0 | 1 | 0 | 18 | 2 | 1 | 2 | 30 | 46 |
| **0,5-1** | 3 | 17 | 22 | 208 | 36 | 45 | 12 | 123 | 168 |
| **1-1,5** | 5 | 33 | 29 | 145 | 66 | 86 | 25 | 131 | 168 |
| **1,5-2** | 15 | 24 | 20 | 83 | 70 | 74 | 23 | 59 | 99 |
| **2-2,5** | 15 | 30 | 20 | 36 | 57 | 64 | 14 | 27 | 56 |
| **2,5-3** | 12 | 25 | 12 | 31 | 46 | 41 | 6 | 14 | 21 |
| **3-3,5** | 7 | 33 | 6 | 26 | 34 | 37 | 5 | 13 | 12 |
| **3,5-4** | 6 | 20 | 17 | 22 | 24 | 26 | 3 | 10 | 12 |
| **4-4,5** | 4 | 19 | 17 | 20 | 20 | 10 | 0 | 4 | 12 |
| **4,5-5** | 7 | 18 | 3 | 12 | 14 | 14 | 2 | 3 | 5 |
| **5-5,5** | 0 | 8 | 8 | 14 | 20 | 12 | 1 | 2 | 7 |
| **5,5-6** | 1 | 13 | 5 | 4 | 10 | 5 | 1 | 1 | 6 |
| **6-6,5** | 3 | 9 | 3 | 7 | 6 | 7 | 1 | 0 | 5 |
| **6,5-7** | 2 | 8 | 1 | 4 | 5 | 10 | 1 | 0 | 1 |
| **7-7,5** | 1 | 10 | 3 | 5 | 7 | 8 | 0 | 1 | 2 |
| **7,5-8** | 0 | 8 | 7 | 3 | 3 | 6 | 0 | 0 | 3 |
| **8-8,5** | 3 | 7 | 2 | 4 | 4 | 5 | 0 | 0 | 3 |
| **8,5-9** | 0 | 5 | 2 | 2 | 6 | 2 | 0 | 0 | 2 |
| **9-9,5** | 0 | 2 | 1 | 2 | 4 | 2 | 1 | 0 | 1 |
| **9,5-10** | 0 | 4 | 1 | 2 | 3 | 2 | 0 | 0 | 1 |
| **≥ 10** | 6 | 19 | 8 | 1 | 18 | 15 | 0 | 0 | 1 |
