# Supplementary material for: Household storage, surplus and supra-household storage in prehistoric and protohistoric societies of the Western Mediterranean
Source: PLoS One. 2020 Sep 14;15(9):e0238237. doi: 10.1371/journal.pone.0238237 (PMC7489512; doi:10.1371/journal.pone.0238237)
Supplement: S3 Table — (DOCX) [file pone.0238237.s004.docx]

**S3 Table. Silo-pit list, chronology (* see Table 1), site name and volumetric calculation.**

| CHRONO* | **SITE** | **NAME SILO-PIT** | **CAPACITY** |
| --- | --- | --- | --- |
| EN | Can Filuà | CF07-24 | 312 |
| EN | Can Filuà | CF07-20 | 785 |
| EN | Can Filuà | CF07-4 | 1911 |
| EN | Can Gambús 1 | 506 | 1504 |
| EN | Coll Blanc | UE 1011 | 1037 |
| EN | El Cavet | 2009 | 190 |
| EN | El Cavet | 2009d | 358 |
| EN | El Cavet | 110 | 385 |
| EN | El Cavet | 2007 | 446 |
| EN | El Cavet | 2008 | 452 |
| EN | El Cavet | 108 | 510 |
| EN | El Cavet | 2012 | 665 |
| EN | El Cavet | 2016 | 726 |
| EN | El Cavet | 117 | 942 |
| EN | El Cavet | 2014 | 1056 |
| EN | Espina C | E-3 | 178 |
| EN | Espina C | E-7 | 313 |
| EN | Font del Ros | EE36 | 201 |
| EN | Font del Ros | EE34 | 254 |
| EN | Font del Ros | EE24 | 318 |
| EN | Font del Ros | EE45 | 380 |
| EN | Font del Ros | EE43 | 192 |
| EN | Font del Ros | EE29 | 241 |
| EN | Font del Ros | EE40 | 319 |
| EN | Font del Ros | EE27 | 320 |
| EN | Font del Ros | EE9 | 350 |
| EN | Font del Ros | EE33 | 550 |
| EN | Font del Ros | EE41 | 557 |
| EN | Font del Ros | EE38 | 681 |
| EN | Font del Ros | EE32 | 919 |
| EN | La Serreta | E-61 | 254 |
| EN | La Serreta | E-75 | 627 |
| EN | Mas d’en Boixos-1 | 299 | 487 |
| EN | Mas d’en Boixos-1 | 338 | 520 |
| EN | Mas d’en Boixos-1 | 327 | 601 |
| EN | Mas d’en Boixos-1 | 303 | 1403 |
| EN | Molins de la Vila | 6149 | 881 |
| EN | Pla de la Bruguera | E8 | 523 |
| EN | Pla del Serrador | E-203 | 353 |
| EN | Pou Nou-2 | E16 | 349 |
| EN | Pou Nou-2 | E11 | 510 |
| EN | Pou Nou-2 | E17 | 602 |
| MN | Mas Vilalba | MV-84 | 56 |
| MN | Pinetons II | E-47 | 100 |
| MN | Les Goges | F7 | 138 |
| MN | Molins de la Vila | 6099 | 154 |
| MN | Can Sadurní | 3 | 178 |
| MN | La Serreta | E-30 | 192 |
| MN | Molins de la Vila | 6147 | 231 |
| MN | Aeroport de Reus | 2022 | 238 |
| MN | El Pujolet de Moja | E6 | 251 |
| MN | La Serreta | E-83 | 255 |
| MN | Bòbila Madurell-Mas Duran | J.24 | 262 |
| MN | El Collet | E-3 | 268 |
| MN | Mas d’en Boixos-1 | 440 | 273 |
| MN | La Serreta | E-26 | 292 |
| MN | Els Mallols | E-227 | 294 |
| MN | Camp de Mas Figueres | UE 204 | 298 |
| MN | Mas Pujó | E-18 | 310 |
| MN | Quatre Pilans I | 1008 | 314 |
| MN | La Serreta | E-9 | 318 |
| MN | El Pujolet de Moja | E15 | 320 |
| MN | La Serreta | E-32 | 323 |
| MN | La Serreta | E-35 | 327 |
| MN | Pou Nou-2 | E12 | 332 |
| MN | La Serreta | E-11 | 341 |
| MN | Aeroport de Reus | 2029 | 341 |
| MN | La Serreta | E-42 | 341 |
| MN | Can Fatjó dels Aurons | 5 | 342 |
| MN | Aeroport de Reus | 125 | 348 |
| MN | Cova de la Pólvora | Fossa 2 | 348 |
| MN | Cova de la Pólvora | Fossa 3 | 348 |
| MN | Mas d’en Boixos-1 | 351 | 349 |
| MN | La Serreta | E-33 | 350 |
| MN | La Serreta | E-28 | 363 |
| MN | Els Cirerers | E17 | 366 |
| MN | Mas d’en Boixos-1 | E-151 | 371 |
| MN | La Serreta | E-34 | 381 |
| MN | Quatre Pilans I | 1010 | 397 |
| MN | Feixa del Moro | N-2 | 398 |
| MN | Cinc Ponts | E-101 | 402 |
| MN | Mas Pujó | E-3 | 405 |
| MN | Mas d’en Boixos-1 | 397 | 418 |
| MN | Mas d’en Boixos-1 | E142 | 428 |
| MN | El Pujolet de Moja | E38d | 435 |
| MN | Camp del Colomer | Fossa 34 | 446 |
| MN | Aeroport de Reus | 298 | 452 |
| MN | Bòbila Madurell-Mas Duran | M.24 | 452 |
| MN | Can Fatjó dels Aurons | 89 | 459 |
| MN | Mas d’en Boixos-1 | 340 | 462 |
| MN | Mas d’en Boixos-1 | 304 | 467 |
| MN | Aeroport de Reus | 2031 | 491 |
| MN | Quatre Pilans I | 1004 | 492 |
| MN | Feixa del Moro | N-3 | 512 |
| MN | Can Sadurní | 1 | 523 |
| MN | Les Goges | F3 | 530 |
| MN | Mas d’en Boixos-1 | 406 | 531 |
| MN | Mas Vilalba | MV-85 | 533 |
| MN | Bòbila Madurell-Mas Duran | 7.3 | 542 |
| MN | Bòbila Madurell-Mas Duran | H.4 | 543 |
| MN | Mas d’en Boixos-1 | 432 | 547 |
| MN | Quatre Pilans I | 1002 | 556 |
| MN | Mas d’en Boixos-1 | 427 | 557 |
| MN | Mas d’en Boixos-1 | 433 | 567 |
| MN | Can Sadurní | 2 | 570 |
| MN | Mas d’en Boixos-1 | 424 | 570 |
| MN | Bòbila Madurell-Mas Duran | H.2 | 591 |
| MN | Bòbila Madurell-Mas Duran | G.23 | 610 |
| MN | Mas d’en Boixos-1 | 360 | 616 |
| MN | Bòbila Madurell-Mas Duran | G.22 | 617 |
| MN | Mas d’en Boixos-1 | 420 | 624 |
| MN | Feixa del Moro | N-1 | 626 |
| MN | Mas d’en Boixos-1 | 362 | 634 |
| MN | Camp del Colomer | Sitja 31 | 637 |
| MN | Mas Pujó | E-26 | 651 |
| MN | Aeroport de Reus | 206 | 655 |
| MN | Camp del Colomer | Sitja 10 | 660 |
| MN | Els Mallols | E-114 | 663 |
| MN | Camí de Cal Piques | E1 | 677 |
| MN | Mas d’en Boixos-1 | 323 | 684 |
| MN | El Pujolet de Moja | E18 | 692 |
| MN | Bòbila Madurell-Mas Duran | 7.4 | 702 |
| MN | Can Roqueta II (est) | 421 | 712 |
| MN | La Serreta | E-69 | 715 |
| MN | Banys de la Mercè | E14 | 723 |
| MN | La Serreta | E-10 | 745 |
| MN | Mas d’en Boixos-1 | 390 | 753 |
| MN | Mas d’en Boixos-1 | 339 | 759 |
| MN | Mas d’en Boixos-1 | 429 | 760 |
| MN | Camp del Colomer | Fossa 14 | 777 |
| MN | Mas d’en Boixos-1 | 333 | 795 |
| MN | Can Fatjó dels Aurons | 6 | 796 |
| MN | Bòbila Madurell-Mas Duran | 11.8 | 800 |
| MN | Mas d’en Boixos-1 | 405 | 803 |
| MN | La Serreta | E-25 | 827 |
| MN | Els Cirerers | E16 | 831 |
| MN | La Serreta | E-56 | 850 |
| MN | Mas d’en Boixos-1 | 418 | 855 |
| MN | Aeroport de Reus | 2013 | 869 |
| MN | Bòbila Madurell-Can Feu | D55 | 878 |
| MN | Mas d’en Boixos-1 | 409 | 918 |
| MN | Mas d’en Boixos-1 | 385 | 923 |
| MN | Saus II/Camp d’en Dalmau | Sitja 36 | 929 |
| MN | Pou Nou-2 | E9 | 930 |
| MN | Camp del Colomer | Sitja 8 | 959 |
| MN | Horts de Can Torras | E11 | 978 |
| MN | La Serreta | E-89 | 980 |
| MN | Bòbila Madurell-Mas Duran | M.23 | 987 |
| MN | Mas d’en Boixos-1 | 412 | 1060 |
| MN | Cova de la Pólvora | Fossa 1 | 1072 |
| MN | Els Mallols | E-228 | 1085 |
| MN | Bòbila Madurell-Mas Duran | J.13 | 1091 |
| MN | Can Roqueta/Torre-Romeu | E66 | 1105 |
| MN | Mas d’en Boixos-1 | E-202 | 1109 |
| MN | La Serreta | E-12 | 1127 |
| MN | Aeroport de Reus | 270 | 1132 |
| MN | Camp del Colomer | Sitja 7 | 1141 |
| MN | Can Roqueta/Torre-Romeu | E195 | 1172 |
| MN | Mas d’en Boixos-1 | E139 | 1238 |
| MN | La Serreta | E-82 | 1242 |
| MN | Molins de la Vila | 6123 | 1250 |
| MN | Mas Pujó | E-17 | 1297 |
| MN | Mas d’en Boixos-1 | 413 | 1348 |
| MN | Mas Vilalba | MV-52 | 1350 |
| MN | Can Roqueta/Torre-Romeu | E189 | 1399 |
| MN | Mas d’en Boixos-1 | E-201 | 1408 |
| MN | Camp del Colomer | Sitja 19 | 1424 |
| MN | Mas d’en Boixos-1 | 311 | 1470 |
| MN | Mas d’en Boixos-1 | 423 | 1512 |
| MN | La Serreta | E-36 | 1574 |
| MN | La Serreta | E-27 | 1656 |
| MN | El Collet | E-5 | 1686 |
| MN | El Collet | E-1 | 1740 |
| MN | Mas d’en Boixos-1 | E136 | 1808 |
| MN | Camp del Colomer | Sitja 1 | 1875 |
| MN | El Collet | E-2 | 1888 |
| MN | Mas d’en Boixos-1 | 417 | 1956 |
| MN | Carrer de les Beates, 2-5 | Sitja | 1986 |
| MN | El Pujolet de Moja | E13 | 2059 |
| MN | Carrers Reina Amàlia 31-33, Lleialtat 1-9 i Carretes 46 i 58 | E-IV | 2297 |
| MN | Camp del Colomer | Sitja 24 | 2415 |
| MN | Mas d’en Boixos-1 | E140 | 2421 |
| MN | Carrers Reina Amàlia 31-33, Lleialtat 1-9 i Carretes 46 i 58 | E-XII | 2462 |
| MN | Carrers Reina Amàlia 31-33, Lleialtat 1-9 i Carretes 46 i 58 | E-XVIII | 2560 |
| LN | Aiguacuit | Estructura 155 | 1259 |
| LN | Aiguacuit | Estructura 137 | 1765 |
| LN | Antic Vapor Gorina | Cubeta 2 | 320 |
| LN | Bòbila Madurell-Mas Duran | I.7 | 445 |
| LN | Bòbila Madurell-Mas Duran | I.8 | 496 |
| LN | Bòbila Madurell-Mas Duran | I.9 | 727 |
| LN | Cal Jardiner I | CJ·I-3 | 430 |
| LN | Camí de Santa Maria dels Horts | E1 | 1051 |
| LN | Camp del Rector | E-2 | 284 |
| LN | Can Gambús 1 | 731 | 327 |
| LN | Can Gambús 1 | 560 | 354 |
| LN | Can Gambús 1 | 569 | 393 |
| LN | Can Gambús 1 | 720 | 453 |
| LN | Can Gambús 1 | 544 | 475 |
| LN | Can Gambús 1 | 732 | 527 |
| LN | Can Gambús 1 | 559 | 584 |
| LN | Can Gambús 1 | 596 | 687 |
| LN | Can Gambús 1 | 499 | 694 |
| LN | Can Gambús 1 | 594 | 705 |
| LN | Can Gambús 1 | 728 | 804 |
| LN | Can Gambús 1 | 502 | 862 |
| LN | Can Gambús 1 | 530 | 954 |
| LN | Can Gambús 1 | 595 | 1149 |
| LN | Can Gambús 1 | 485 | 1326 |
| LN | Can Gambús 1 | 579 | 1895 |
| LN | Can Gambús 1 | 582 | 2094 |
| LN | Cantorella | FC-12 | 1988 |
| LN | Cantorella | FC-185 | 145 |
| LN | Cantorella | FC-196 | 347 |
| LN | Cantorella | FC-198 | 255 |
| LN | Cantorella | FC-207 | 238 |
| LN | Cantorella | FC-216 | 326 |
| LN | Cantorella | FC-222 | 312 |
| LN | Cantorella | FC-224 | 678 |
| LN | Cantorella | FC-238 | 251 |
| LN | Cantorella | FC-3 | 826 |
| LN | Cantorella | FC-9 | 513 |
| LN | Cantorella | SJ-10 | 849 |
| LN | Cantorella | SJ-13 | 3145 |
| LN | Cantorella | SJ-134 | 1659 |
| LN | Cantorella | SJ-156 | 603 |
| LN | Cantorella | SJ-157 | 411 |
| LN | Cantorella | SJ-178 | 318 |
| LN | Cantorella | SJ-182 | 271 |
| LN | Cantorella | SJ-186 | 1181 |
| LN | Cantorella | SJ-187 | 602 |
| LN | Cantorella | SJ-188 | 1377 |
| LN | Cantorella | SJ-191 | 1541 |
| LN | Cantorella | SJ-192 | 649 |
| LN | Cantorella | SJ-194 | 375 |
| LN | Cantorella | SJ-202 | 337 |
| LN | Cantorella | SJ-208 | 905 |
| LN | Cantorella | SJ-209 | 1181 |
| LN | Cantorella | SJ-217 | 1057 |
| LN | Cantorella | SJ-218 | 779 |
| LN | Cantorella | SJ-228 | 496 |
| LN | Cantorella | SJ-236 | 238 |
| LN | Cantorella | SJ-240 | 900 |
| LN | Cantorella | SJ-241 | 855 |
| LN | Cantorella | SJ-242 | 1214 |
| LN | Cantorella | SJ-258 | 565 |
| LN | Cantorella | SJ-260 | 1041 |
| LN | Cantorella | SJ-274 | 288 |
| LN | Cantorella | SJ-295 | 399 |
| LN | Cantorella | SJ-328 | 1063 |
| LN | Cantorella | SJ-36 | 306 |
| LN | Cantorella | SJ-63 | 3672 |
| LN | Cinc Ponts | E-94 | 796 |
| LN | Cinc Ponts | E-102 | 1095 |
| LN | Cinc Ponts | E-41 | 1139 |
| LN | Cinc Ponts | E-67 | 1380 |
| LN | Cinc Ponts | E-44 | 1896 |
| LN | Coll Blanc | UE 1080 | 122 |
| LN | Els Pujols/Serra de la Pairal | E1 | 571 |
| LN | Espina C | E-14 | 452 |
| LN | Espina C | E-27 | 542 |
| LN | Espina C | E-13 | 564 |
| LN | Espina C | E-4 | 589 |
| LN | Espina C | E-10 | 595 |
| LN | Espina C | E-28 | 631 |
| LN | Espina C | E-5 | 991 |
| LN | Espina C | E-2 | 1085 |
| LN | Espina C | E-15 | 1133 |
| LN | Espina C | E-9 | 1697 |
| LN | La Serreta | E-70 | 415 |
| LN | Mas d’en Boixos-1 | 295 | 110 |
| LN | Mas d’en Boixos-1 | E143 | 238 |
| LN | Mas d’en Boixos-1 | E-149 | 266 |
| LN | Mas d’en Boixos-1 | E141 | 433 |
| LN | Mas d’en Boixos-1 | E144 | 452 |
| LN | Mas d’en Boixos-1 | E-200 | 454 |
| LN | Mas d’en Boixos-1 | 316 | 575 |
| LN | Mas d’en Boixos-1 | 395 | 942 |
| LN | Masia Can Roqueta-Edifici Annex | 2 | 923 |
| LN | Masia Can Roqueta-Edifici Annex | 8 | 1130 |
| LN | Minferri | 124 | 953 |
| LN | Minferri | 89 | 1970 |
| LN | Minferri | 191 | 3961 |
| LN | Serrat dels Espinyers | 1078 | 3322 |
| LN | Sindreu 1 | PS1/1 | 2661 |
| LN | Sindreu 1 | PS1/2 | 3213 |
| LN | Sindreu 1 | PS1/6 | 3820 |
| EBA | Minferri | 261 | 33 |
| EBA | Minferri | 126 | 71 |
| EBA | Minferri | 319 | 73 |
| EBA | Minferri | 19 | 89 |
| EBA | Cinc Ponts | E-88 | 103 |
| EBA | Minferri | 210 | 109 |
| EBA | Minferri | 271 | 110 |
| EBA | Minferri | 309 | 112 |
| EBA | Can Gambús 1 | 718 | 113 |
| EBA | Minferri | 16 | 116 |
| EBA | Minferri | 304 | 122 |
| EBA | Minferri | 252 | 127 |
| EBA | Minferri | 249 | 128 |
| EBA | Minferri | 267 | 133 |
| EBA | Pla de Tabac I i II | UE 2400 | 146 |
| EBA | Minferri | 287 | 154 |
| EBA | Can Sant Joan | 14 | 157 |
| EBA | Cantorella | SJ-257 | 159 |
| EBA | Can Roqueta II (est) | 529 | 180 |
| EBA | Minferri | 113 | 195 |
| EBA | Minferri | 219 | 201 |
| EBA | Minferri | 92 | 211 |
| EBA | Cantorella | SJ-133 | 217 |
| EBA | Cinc Ponts | E-91 | 220 |
| EBA | Bòbila Madurell-Can Feu | D14 | 223 |
| EBA | Pla de Tabac I i II | UE 2700 | 227 |
| EBA | Minferri | 189 | 235 |
| EBA | La Girada | E23 | 243 |
| EBA | Can Roqueta II (est) | 506 | 249 |
| EBA | Minferri | 187 | 250 |
| EBA | Minferri | 114 | 262 |
| EBA | Minferri | 246 | 269 |
| EBA | Minferri | 370 | 273 |
| EBA | Minferri | 377 | 278 |
| EBA | Minferri | 403 | 285 |
| EBA | Minferri | 311 | 289 |
| EBA | Cantorella | SJ-195 | 303 |
| EBA | Sitges de la UAB | E-26 | 303 |
| EBA | Minferri | 160 | 309 |
| EBA | Can Roqueta II (est) | 592 | 310 |
| EBA | Cova Colomera | EE1 | 314 |
| EBA | Llirians del Mas, Les Torres i Sector Rec | SL-7 | 322 |
| EBA | Cinc Ponts | E-72 | 328 |
| EBA | Minferri | 244 | 332 |
| EBA | Vinya del Corb | E-4 | 332 |
| EBA | Minferri | 178 | 340 |
| EBA | Can Roqueta II (est) | 618 | 343 |
| EBA | Minferri | 323 | 344 |
| EBA | Minferri | 51 | 358 |
| EBA | Minferri | 299 | 358 |
| EBA | Can Roqueta/Torre-Romeu | E3 | 363 |
| EBA | Minferri | 384 | 369 |
| EBA | Can Roqueta II (est) | 549 | 371 |
| EBA | Can Roqueta II (est) | 737 | 372 |
| EBA | Minferri | 410 | 375 |
| EBA | Aeroport de Reus | 2033 | 385 |
| EBA | Minferri | 226 | 389 |
| EBA | Minferri | 375 | 390 |
| EBA | Minferri | 119 | 397 |
| EBA | Minferri | 229 | 397 |
| EBA | Minferri | 71 | 398 |
| EBA | Can Roqueta II (est) | 571 | 400 |
| EBA | Minferri | 341 | 401 |
| EBA | Cantorella | SJ-139 | 402 |
| EBA | Can Roqueta/Torre-Romeu | E30 | 408 |
| EBA | Minferri | 368 | 415 |
| EBA | Can Roqueta II (est) | 657 | 416 |
| EBA | Can Roqueta II (est) | 656 | 416 |
| EBA | Can Roqueta II (est) | 533 | 417 |
| EBA | Camp Cinzano | E-18 | 435 |
| EBA | Pla de Tabac I i II | UE 3400 | 440 |
| EBA | Can Gambús 2 | UE 201 | 442 |
| EBA | Can Gambús 1 | 142 | 443 |
| EBA | Can Filuà | E-VI | 444 |
| EBA | Cinc Ponts | E-78 | 453 |
| EBA | Minferri | 238 | 453 |
| EBA | Can Roqueta II (est) | 246 | 458 |
| EBA | Pla de Tabac I i II | UE 3000 | 463 |
| EBA | Minferri | 177 | 464 |
| EBA | Can Gambús 1 | 714 | 464 |
| EBA | Can Roqueta II (est) | 248 | 464 |
| EBA | Bòbila Madurell-Can Feu | F1 | 474 |
| EBA | Can Roqueta II (est) | 346 | 476 |
| EBA | Minferri | 137 | 480 |
| EBA | Can Sant Joan | 13 | 482 |
| EBA | Can Fatjó dels Aurons | 116 | 486 |
| EBA | Can Roqueta II (est) | 475 | 492 |
| EBA | Turó de la Bateria/Hotel Ibis | E-27 | 501 |
| EBA | La Girada | E25 | 511 |
| EBA | Can Roqueta II (est) | 639 | 512 |
| EBA | Can Gambús 2 | UE 834 | 513 |
| EBA | Minferri | 150 | 517 |
| EBA | Pla de Tabac I i II | UE 2500 | 518 |
| EBA | Llirians del Mas, Les Torres i Sector Rec | E-27 | 523 |
| EBA | Can Roqueta II (est) | 481 | 529 |
| EBA | Llirians del Mas, Les Torres i Sector Rec | SL-1 | 534 |
| EBA | Sitges de la UAB | E-20 | 535 |
| EBA | Sant Joan Nord | UE 27 | 541 |
| EBA | Can Gambús 2 | UE 423 | 541 |
| EBA | Can Gambús 1 | 736 | 553 |
| EBA | Minferri | 235 | 553 |
| EBA | Bòbila Madurell-Can Feu | D48 | 555 |
| EBA | Can Roqueta II (est) | 595 | 556 |
| EBA | Minferri | 190 | 564 |
| EBA | Minferri | 197 | 568 |
| EBA | Cinc Ponts | E-125 | 574 |
| EBA | Minferri | 139 | 575 |
| EBA | Minferri | 360 | 591 |
| EBA | Can Roqueta II (est) | 589 | 596 |
| EBA | Can Roqueta II (est) | 756 | 596 |
| EBA | Cantorella | SJ-205 | 597 |
| EBA | Bòbila Madurell-Can Feu | D18 | 604 |
| EBA | Can Filuà | CF07-39 | 604 |
| EBA | Cantorella | SJ-253 | 612 |
| EBA | Minferri | 90 | 617 |
| EBA | Can Roqueta II (est) | 532 | 617 |
| EBA | Can Roqueta II (est) | 468 | 623 |
| EBA | Pla de Tabac I i II | UE 2600 | 623 |
| EBA | Cantorella | SJ-268 | 624 |
| EBA | Minferri | 87 | 625 |
| EBA | Can Roqueta II (est) | 623 | 628 |
| EBA | Minferri | 34 | 630 |
| EBA | Minferri | 374 | 635 |
| EBA | Can Roqueta II (est) | 474 | 637 |
| EBA | Minferri | 233 | 641 |
| EBA | Minferri | 419 | 643 |
| EBA | Can Roqueta II (est) | 621 | 652 |
| EBA | Cantorella | SJ-163 | 658 |
| EBA | Can Roqueta II (est) | 613 | 661 |
| EBA | Minferri | 257 | 668 |
| EBA | Vinya del Corb | E-5 | 670 |
| EBA | Minferri | 362 | 677 |
| EBA | Torrebonica | S-38 | 678 |
| EBA | Minferri | 245 | 678 |
| EBA | Cinc Ponts | E-97 | 687 |
| EBA | Cantorella | SJ-166 | 690 |
| EBA | Can Roqueta II (est) | 866 | 692 |
| EBA | Can Roqueta/Can Revella | E59 | 693 |
| EBA | Can Gambús 1 | 533 | 694 |
| EBA | Minferri | 396 | 694 |
| EBA | Can Roqueta/Can Revella | E137 | 698 |
| EBA | Can Roqueta II (est) | 740 | 698 |
| EBA | Can Roqueta II (est) | 558 | 700 |
| EBA | Can Roqueta II (est) | 653 | 707 |
| EBA | Camps de Can Colomer | UE 938 | 708 |
| EBA | Llirians del Mas, Les Torres i Sector Rec | SL-2 | 709 |
| EBA | Can Gambús 2 | UE 4035 | 718 |
| EBA | Minferri | 373 | 729 |
| EBA | Cantorella | SJ-170 | 730 |
| EBA | Can Gambús 2 | UE 417 | 730 |
| EBA | Cantorella | SJ-237 | 731 |
| EBA | Minferri | 66 | 735 |
| EBA | Pla del Serrador | E-64 | 744 |
| EBA | Minferri | 184 | 746 |
| EBA | Can Roqueta II (est) | 505 | 746 |
| EBA | Llirians del Mas, Les Torres i Sector Rec | E-26 | 753 |
| EBA | Cantorella | SJ-265 | 756 |
| EBA | Pla del Serrador | E-5 | 760 |
| EBA | Can Roqueta II (est) | 460 | 763 |
| EBA | Can Roqueta II (est) | 132 | 764 |
| EBA | Can Roqueta II (est) | 588 | 764 |
| EBA | Cantorella | SJ-141 | 765 |
| EBA | Can Fatjó dels Aurons | 115 | 768 |
| EBA | Can Roqueta-DIASA | 65 | 769 |
| EBA | Minferri | 228 | 772 |
| EBA | Cantorella | SJ-145 | 791 |
| EBA | Minferri | 169 | 794 |
| EBA | Can Roqueta II (est) | 504 | 803 |
| EBA | Can Roqueta/Torre-Romeu | E162 | 807 |
| EBA | Llirians del Mas, Les Torres i Sector Rec | E-17 | 811 |
| EBA | Can Roqueta II (est) | 539 | 822 |
| EBA | Cantorella | SJ-30 | 828 |
| EBA | Minferri | 433 | 831 |
| EBA | Camp Cinzano | E-11 | 835 |
| EBA | Minferri | 148 | 835 |
| EBA | Cantorella | SJ-137 | 848 |
| EBA | Can Roqueta II (est) | 753 | 850 |
| EBA | Can Gambús 1 | 480 | 855 |
| EBA | Bòbila Madurell-Can Feu | E11 | 858 |
| EBA | Cantorella | SJ-158 | 858 |
| EBA | Minferri | 10 | 858 |
| EBA | Mercat de Santa Caterina | UE 10017 | 859 |
| EBA | Minferri | 32 | 866 |
| EBA | Minferri | 153 | 874 |
| EBA | Can Roqueta II (est) | 557 | 879 |
| EBA | Camp Cinzano | E-24 | 883 |
| EBA | Aeroport de Reus | 4008 | 887 |
| EBA | Mercat de Santa Caterina | UE 10018 | 890 |
| EBA | Can Roqueta II (est) | 243 | 890 |
| EBA | Minferri | 294 | 904 |
| EBA | Can Roqueta II (est) | 607 | 906 |
| EBA | Pla de Tabac I i II | UE 1000 | 907 |
| EBA | Can Roqueta-DIASA | 68 | 909 |
| EBA | Can Roqueta II (est) | 49 | 911 |
| EBA | Minferri | 130 | 916 |
| EBA | Minferri | 198 | 924 |
| EBA | Can Roqueta II (est) | 44 | 934 |
| EBA | Can Roqueta II (est) | 610 | 936 |
| EBA | Bòbila Madurell-Can Feu | D36 | 942 |
| EBA | Minferri | 44 | 944 |
| EBA | Camp Cinzano | E-14 | 947 |
| EBA | Minferri | 105 | 950 |
| EBA | Minferri | 366 | 951 |
| EBA | Can Roqueta II (est) | 414 | 952 |
| EBA | Can Roqueta II (est) | 858 | 953 |
| EBA | Can Roqueta II (est) | 614 | 953 |
| EBA | Sant Joan Nord | UE 122 | 955 |
| EBA | Can Roqueta II (est) | 68 | 956 |
| EBA | Minferri | 236 | 956 |
| EBA | Pla de Tabac I i II | UE 1900 | 959 |
| EBA | Minferri | 388 | 961 |
| EBA | Cantorella | SJ-162 | 966 |
| EBA | Can Gambús 1 | 126 | 971 |
| EBA | Minferri | 17 | 980 |
| EBA | Minferri | 183 | 984 |
| EBA | Can Roqueta II (est) | 550 | 985 |
| EBA | Cantorella | SJ-259 | 990 |
| EBA | Minferri | 225 | 1001 |
| EBA | Llirians del Mas, Les Torres i Sector Rec | SL-8 | 1013 |
| EBA | Cantorella | SJ-175 | 1020 |
| EBA | Sitges de la UAB | E-25 | 1020 |
| EBA | Minferri | 188 | 1021 |
| EBA | Minferri | 69 | 1021 |
| EBA | Bòbila Madurell-Can Feu | F2 | 1026 |
| EBA | Can Filuà | CF07-42 | 1029 |
| EBA | Minferri | 68 | 1038 |
| EBA | Mercat de Santa Caterina | UE 5504 | 1040 |
| EBA | Camp Cinzano | E-7 | 1041 |
| EBA | Can Roqueta II (est) | 131 | 1045 |
| EBA | Cantorella | SJ-142 | 1049 |
| EBA | Minferri | 428 | 1049 |
| EBA | Minferri | 289 | 1052 |
| EBA | Minferri | 292 | 1056 |
| EBA | Mas d’en Boixos-1 | 431 | 1059 |
| EBA | Minferri | 369 | 1059 |
| EBA | Sitja del Camí del Mig | E-1 | 1063 |
| EBA | Can Roqueta II (est) | 43 | 1063 |
| EBA | Minferri | 54 | 1075 |
| EBA | Can Roqueta II (est) | 520 | 1081 |
| EBA | Minferri | 202 | 1083 |
| EBA | Cantorella | SJ-138 | 1085 |
| EBA | Pla de Tabac I i II | UE 1200 | 1101 |
| EBA | Can Filuà | CF07-38 | 1101 |
| EBA | Can Roqueta II (est) | 647 | 1109 |
| EBA | Cantorella | SJ-172 | 1119 |
| EBA | Can Roqueta II (est) | 583 | 1120 |
| EBA | Camp Cinzano | E-16 | 1121 |
| EBA | Can Roqueta II (est) | 640 | 1126 |
| EBA | Els Cirerers | E11 | 1128 |
| EBA | Sant Llorenç de Boada-el Graell | S-32 | 1130 |
| EBA | Can Roqueta/Torre-Romeu | E191 | 1132 |
| EBA | Minferri | 64 | 1134 |
| EBA | Minferri | 401 | 1141 |
| EBA | Can Roqueta II (est) | 648 | 1146 |
| EBA | Minferri | 59 | 1155 |
| EBA | Bòbila Madurell-Can Feu | E27 | 1159 |
| EBA | Can Roqueta II (est) | 628 | 1168 |
| EBA | Minferri | 310 | 1172 |
| EBA | Minferri | 376 | 1176 |
| EBA | Aeroport de Reus | 4006 | 1177 |
| EBA | Minferri | 175 | 1184 |
| EBA | Cantorella | SJ-159 | 1191 |
| EBA | Can Roqueta II (est) | 736 | 1200 |
| EBA | Can Roqueta II (est) | 654 | 1203 |
| EBA | Can Roqueta II (est) | 586 | 1208 |
| EBA | Institut de Batxillerat Antoni Pous/Institut de Manlleu | IM-15 | 1212 |
| EBA | Minferri | 180 | 1214 |
| EBA | Can Roqueta II (est) | 46 | 1215 |
| EBA | Institut de Batxillerat Antoni Pous/Institut de Manlleu | IM-31 | 1227 |
| EBA | Minferri | 381 | 1227 |
| EBA | Can Roqueta II (est) | 622 | 1231 |
| EBA | Camp Cinzano | E-15 | 1245 |
| EBA | Can Roqueta II (est) | 590 | 1246 |
| EBA | Can Roqueta II (est) | 483 | 1248 |
| EBA | Minferri | 262 | 1256 |
| EBA | Minferri | 430 | 1264 |
| EBA | Minferri | 243 | 1264 |
| EBA | Pla de Tabac I i II | UE 2300 | 1270 |
| EBA | Cantorella | SJ-146 | 1273 |
| EBA | Bòbila Madurell-Can Feu | E16 | 1273 |
| EBA | Carrer Riereta, 37-37 bis/Sant Pau, 84 | E-16b | 1281 |
| EBA | Minferri | 156 | 1282 |
| EBA | Can Gambús 2 | UE 449 | 1285 |
| EBA | Minferri | 395 | 1288 |
| EBA | Cantorella | SJ-29 | 1298 |
| EBA | Cantorella | SJ-169 | 1305 |
| EBA | Institut de Batxillerat Antoni Pous/Institut de Manlleu | IM-20 | 1309 |
| EBA | Pla del Serrador | E-74 | 1323 |
| EBA | Carrer Ca n’Alzina - Carrer del Mas Carbó (Can Roqueta) | E-2 | 1329 |
| EBA | Minferri | 53 | 1329 |
| EBA | Can Roqueta II (est) | 743 | 1335 |
| EBA | Can Roqueta II (est) | 594 | 1344 |
| EBA | Can Ballarà | CB 1 | 1358 |
| EBA | Can Roqueta/Torre-Romeu | E7 | 1368 |
| EBA | Camp Cinzano | E-6 | 1379 |
| EBA | Can Roqueta II (est) | 348 | 1390 |
| EBA | Can Filuà | CF07-29 | 1392 |
| EBA | Minferri | 203 | 1400 |
| EBA | Bòbila Madurell-Can Feu | D19 | 1402 |
| EBA | Minferri | 193 | 1435 |
| EBA | Can Roqueta II (est) | 442 | 1464 |
| EBA | Carrer Riereta, 37-37 bis/Sant Pau, 84 | E-16a | 1477 |
| EBA | Minferri | 194 | 1482 |
| EBA | Minferri | 155 | 1497 |
| EBA | Sitges de la UAB | E-35 | 1504 |
| EBA | Minferri | 128 | 1505 |
| EBA | Can Roqueta II (est) | 556 | 1509 |
| EBA | Carrer Ca n’Alzina - Carrer del Mas Carbó (Can Roqueta) | E-3 | 1519 |
| EBA | Minferri | 168 | 1523 |
| EBA | Minferri | 383 | 1530 |
| EBA | Minferri | 382 | 1592 |
| EBA | Can Gambús 2 | UE 451 | 1601 |
| EBA | Minferri | 380 | 1645 |
| EBA | Minferri | 55 | 1647 |
| EBA | Can Roqueta II (est) | 626 | 1654 |
| EBA | Can Roqueta II (est) | 325 | 1673 |
| EBA | Can Roqueta II (est) | 220 | 1681 |
| EBA | Minferri | 286 | 1684 |
| EBA | Can Roqueta II (est) | 627 | 1684 |
| EBA | Minferri | 402 | 1695 |
| EBA | Can Gambús 1 | 563 | 1709 |
| EBA | Can Roqueta II (est) | 591 | 1711 |
| EBA | Estació de bombeig EDAR Sabadell-riu Ripoll (Can Roqueta) | CR-148 | 1712 |
| EBA | Can Roqueta II (est) | 634 | 1716 |
| EBA | Minferri | 352 | 1719 |
| EBA | Minferri | 38 | 1736 |
| EBA | Minferri | 400 | 1762 |
| EBA | Minferri | 275 | 1785 |
| EBA | Can Roqueta | E-1 Nord | 1786 |
| EBA | Can Roqueta | E-1 Sud | 1786 |
| EBA | Cantorella | SJ-31 | 1790 |
| EBA | Minferri | 331 | 1823 |
| EBA | Minferri | 364 | 1857 |
| EBA | Minferri | 67 | 1862 |
| EBA | Can Roqueta II (est) | 536 | 1864 |
| EBA | Minferri | 417 | 1879 |
| EBA | Can Roqueta II (est) | 759 | 1889 |
| EBA | Can Roqueta II (est) | 644 | 1918 |
| EBA | Pla de Tabac I i II | UE 2900 | 1929 |
| EBA | Minferri | 435 | 1960 |
| EBA | Cinc Ponts | E-95 | 2000 |
| EBA | Minferri | 88 | 2033 |
| EBA | Can Roqueta II (est) | 221 | 2067 |
| EBA | Minferri | 359 | 2073 |
| EBA | Cantorella | SJ-28 | 2076 |
| EBA | Can Roqueta II (est) | 645 | 2086 |
| EBA | Can Gambús 1 | 143 | 2096 |
| EBA | Can Roqueta II (est) | 498 | 2114 |
| EBA | Minferri | 385 | 2135 |
| EBA | Minferri | 31 | 2145 |
| EBA | Llirians del Mas, Les Torres i Sector Rec | E-28 | 2161 |
| EBA | Minferri | 398 | 2173 |
| EBA | Can Roqueta II (est) | 463 | 2200 |
| EBA | Minferri | 354 | 2202 |
| EBA | Minferri | 386 | 2208 |
| EBA | Can Roqueta/Torre-Romeu | E10 | 2237 |
| EBA | Cantorella | SJ-27 | 2378 |
| EBA | Can Roqueta II (est) | 354 | 2390 |
| EBA | Cantorella | SJ-47 | 2402 |
| EBA | Can Roqueta II (est) | 36 | 2502 |
| EBA | Minferri | 429 | 2505 |
| EBA | Minferri | 431 | 2533 |
| EBA | Cantorella | SJ-132 | 2608 |
| EBA | Minferri | 361 | 2623 |
| EBA | Minferri | 222 | 2653 |
| EBA | Minferri | 353 | 2691 |
| EBA | Cinc Ponts | E-103 | 2713 |
| EBA | Minferri | 136 | 2726 |
| EBA | Bòbila Madurell-Can Feu | E2b | 2769 |
| EBA | Minferri | 405 | 2930 |
| EBA | Minferri | 95 | 3122 |
| EBA | Cantorella | SJ-177 | 3123 |
| EBA | Minferri | 367 | 3268 |
| EBA | Minferri | 391 | 3499 |
| EBA | Minferri | 248 | 3544 |
| EBA | Minferri | 161 | 3585 |
| EBA | Can Filuà | PCT2 | 3607 |
| EBA | Minferri | 399 | 3692 |
| EBA | Minferri | 425 | 3714 |
| EBA | Minferri | 372 | 3718 |
| EBA | Can Roqueta II (est) | 436 | 3780 |
| EBA | Minferri | 418 | 3815 |
| EBA | Cantorella | SJ-42 | 3848 |
| EBA | Can Soldevila | CS V | 3907 |
| EBA | Can Roqueta II (est) | 738 | 3949 |
| EBA | Minferri | 227 | 4050 |
| EBA | Minferri | 355 | 4069 |
| EBA | Cantorella | SJ-43 | 4852 |
| EBA | Can Roqueta II (est) | 499 | 4980 |
| EBA | Can Filuà | PCT1 | 6423 |
| EBA | Minferri | 86 | 7066 |
| LBA | Can Roqueta/Can Revella | E245 | 10 |
| LBA | Can Roqueta/Can Revella | E19 | 221 |
| LBA | Pinetons I | E-76 | 250 |
| LBA | Carrer Can Camps/Avinguda Can Bordoll (Can Roqueta) | CR-140 | 305 |
| LBA | Torre Vedruna-Sector Transports | E-62 | 460 |
| LBA | Escola bressol de la Florida Nord | UE 2 | 495 |
| LBA | Santa Digna III | 3001 | 566 |
| LBA | Can Roqueta/Can Revella | E102 | 567 |
| LBA | Santa Digna III | 3003 | 606 |
| LBA | Can Roqueta/Can Revella | E108 | 637 |
| LBA | Can Roqueta II (est) | 601 | 667 |
| LBA | Can Roqueta II (est) | 820 | 759 |
| LBA | Can Roqueta-DIASA | 128 | 838 |
| LBA | Santa Digna III | 3009 | 897 |
| LBA | Can Roqueta-DIASA | 42 | 906 |
| LBA | Santa Digna III | 3005 | 958 |
| LBA | Can Roqueta-DIASA | 41 | 969 |
| LBA | Can Roqueta II (est) | 304 | 971 |
| LBA | Can Roqueta II (est) | 821 | 1056 |
| LBA | Can Roqueta II (est) | 559 | 1058 |
| LBA | Can Roqueta/Can Revella | E82 | 1064 |
| LBA | Camp d’en Pitu Porusia | FS-3 (1035) | 1079 |
| LBA | Escola bressol de la Florida Nord | UE 4 | 1080 |
| LBA | Can Roqueta/Torre-Romeu | E108 | 1096 |
| LBA | Pinetons II | E-4 | 1165 |
| LBA | Can Roqueta/Torre-Romeu | E194 | 1213 |
| LBA | La Masieta de Queralt | 1 | 1219 |
| LBA | Can Roqueta/Can Revella | E104 | 1244 |
| LBA | Can Roqueta/Torre-Romeu | E118 | 1278 |
| LBA | Can Roqueta II (est) | 322 | 1303 |
| LBA | Can Roqueta/Torre-Romeu | E99 | 1339 |
| LBA | Bòbila Madurell-Mas Duran | K.3 | 1357 |
| LBA | Can Roqueta II (est) | 803 | 1366 |
| LBA | Can Roqueta II (est) | 379 | 1374 |
| LBA | Bosc del Quer | E-178 | 1446 |
| LBA | Escola bressol de la Florida Nord | UE 139 | 1458 |
| LBA | Can Roqueta II (est) | 794 | 1504 |
| LBA | Camp d’en Pitu Porusia | FS-2 (1015) | 1509 |
| LBA | Can Roqueta/Can Revella | E112 | 1559 |
| LBA | Pinetons I | E-84 | 1581 |
| LBA | Can Roqueta/Torre-Romeu | E106 | 1620 |
| LBA | Can Roqueta II (est) | 364 | 1671 |
| LBA | Can Roqueta/Torre-Romeu | E104 | 1706 |
| LBA | Can Roqueta/Torre-Romeu | E111 | 1720 |
| LBA | Bòbila Madurell-Mas Duran | J.3 | 1723 |
| LBA | Can Roqueta II (est) | 662 | 1735 |
| LBA | Can Roqueta II (est) | 778 | 1756 |
| LBA | Can Roqueta/Torre-Romeu | E110 | 1761 |
| LBA | Can Roqueta/Torre-Romeu | E55 | 1769 |
| LBA | Can Roqueta/Can Revella | E120 | 1772 |
| LBA | Pinetons I | E-90 | 1820 |
| LBA | Can Roqueta/Can Revella | E198 | 1905 |
| LBA | Can Roqueta II (est) | 835 | 1914 |
| LBA | Can Roqueta II (est) | 785 | 1927 |
| LBA | Pinetons I | E-79 | 1929 |
| LBA | Can Roqueta II (est) | 826 | 1955 |
| LBA | Bòbila Madurell-Mas Duran | J.2 | 1972 |
| LBA | Bosc del Quer | E-518 | 2014 |
| LBA | Bòbila Madurell-Can Feu | D12b | 2037 |
| LBA | Can Roqueta/Can Revella | E121 | 2042 |
| LBA | Can Roqueta II (est) | 789 | 2058 |
| LBA | Can Roqueta/Can Revella | E187 | 2066 |
| LBA | Can Roqueta II (est) | 793 | 2080 |
| LBA | Bòbila Madurell-Can Feu | D16 | 2089 |
| LBA | Can Roqueta/Torre-Romeu | E217 | 2095 |
| LBA | Can Roqueta/Can Revella | E186 | 2133 |
| LBA | Can Roqueta II (est) | 666 | 2161 |
| LBA | Can Roqueta-DIASA | 81 | 2205 |
| LBA | Can Roqueta/Torre-Romeu | E200 | 2229 |
| LBA | Can Roqueta/Torre-Romeu | E203 | 2234 |
| LBA | Can Roqueta/Torre-Romeu | E267 | 2270 |
| LBA | Can Roqueta/Can Revella | E63 | 2387 |
| LBA | Mas d’en Boixos-1 | 382 | 2432 |
| LBA | Can Roqueta II (est) | 818 | 2445 |
| LBA | Can Soldevila | CS IX | 2469 |
| LBA | Can Roqueta/Torre-Romeu | E218 | 2476 |
| LBA | Pinetons I | E-54 | 2480 |
| LBA | Can Roqueta/Torre-Romeu | E64 | 2496 |
| LBA | Can Roqueta/Torre-Romeu | E228 | 2546 |
| LBA | Can Roqueta/Can Revella | E214 | 2553 |
| LBA | Can Roqueta II (est) | 791 | 2587 |
| LBA | Can Roqueta/Torre-Romeu | E201 | 2591 |
| LBA | Pinetons I | E-88 | 2616 |
| LBA | Can Roqueta/Torre-Romeu | E63 | 2640 |
| LBA | Can Gambús 1 | 448 | 2648 |
| LBA | Can Roqueta II (est) | 797 | 2654 |
| LBA | Can Roqueta II (est) | 127 | 2709 |
| LBA | Can Roqueta/Can Revella | E85 | 2712 |
| LBA | Can Roqueta II (est) | 817 | 2719 |
| LBA | Can Roqueta/Can Revella | E194 | 2720 |
| LBA | Bòbila Madurell-Mas Duran | G.2 | 2725 |
| LBA | Can Gambús 1 | 450 | 2740 |
| LBA | Mas d’en Boixos-1 | 444 | 2761 |
| LBA | Can Roqueta/Can Revella | E111 | 2780 |
| LBA | Can Roqueta-DIASA | 129 | 2836 |
| LBA | Can Roqueta/Can Revella | E184 | 2836 |
| LBA | Can Roqueta/Can Revella | E92 | 2849 |
| LBA | Mas d’en Boixos-1 | 445 | 2853 |
| LBA | Can Roqueta II (est) | 824 | 2898 |
| LBA | Can Roqueta-DIASA | 74 | 2923 |
| LBA | Can Roqueta/Can Revella | E93 | 2959 |
| LBA | Can Roqueta/Torre-Romeu | E224 | 2974 |
| LBA | Can Roqueta/Torre-Romeu | E214 | 3070 |
| LBA | Can Roqueta/Torre-Romeu | E202 | 3103 |
| LBA | Can Roqueta/Can Revella | E173 | 3123 |
| LBA | Can Roqueta II (est) | 790 | 3141 |
| LBA | Can Roqueta II (est) | 816 | 3147 |
| LBA | Can Roqueta/Can Revella | CRCRV294 | 3254 |
| LBA | Bòbila Madurell-Can Feu | D47a | 3294 |
| LBA | Can Roqueta/Can Revella | E234 | 3602 |
| LBA | Can Roqueta/Can Revella | E140 | 3643 |
| LBA | Can Roqueta/Can Revella | E191 | 3711 |
| LBA | Can Roqueta/Can Revella | E128 | 3732 |
| LBA | Can Roqueta II (est) | 813 | 3909 |
| LBA | Can Roqueta/Can Revella | E126 | 4054 |
| LBA | Can Roqueta/Torre-Romeu | E222 | 4071 |
| LBA | Can Roqueta II (est) | 836 | 4402 |
| LBA | Can Roqueta II (est) | 130 | 4699 |
| LBA | Can Roqueta/Can Revella | E110 | 4729 |
| EIA | Mas d’en Boixos-1 | 371 | 57 |
| EIA | L’Era del Castell | 1505 | 216 |
| EIA | Vil·la romana de Sant Amanç | UE 4 | 274 |
| EIA | Can Roqueta/Can Revella | E117 | 397 |
| EIA | L’Era del Castell | 1504 | 482 |
| EIA | Can Roqueta II (est) | 763 | 503 |
| EIA | Pou Nou-2 | E2 | 539 |
| EIA | Can Gambús 1 | 723 | 647 |
| EIA | Can Roqueta/Can Revella | E44 | 665 |
| EIA | Can Roqueta II (est) | 711 | 698 |
| EIA | Can Roqueta II (est) | 365 | 720 |
| EIA | Can Gambús 1 | 722 | 729 |
| EIA | Can Roqueta-DIASA | 98 | 809 |
| EIA | Can Gambús 1 | 750 | 810 |
| EIA | Can Roqueta/Can Revella | CRCRV325 | 818 |
| EIA | Camp Gran | UE 3121 | 849 |
| EIA | Can Roqueta II (est) | 745 | 861 |
| EIA | Mas d’en Boixos-1 | 402 | 870 |
| EIA | Els Vilars | SJ-951 | 896 |
| EIA | Mas d’en Boixos-1 | E-153 | 904 |
| EIA | Els Vilars | SJ-952 | 959 |
| EIA | Can Roqueta II (est) | 363 | 964 |
| EIA | Sitges de la UAB | E-1 | 986 |
| EIA | Can Roqueta II (est) | 374 | 1058 |
| EIA | Can Gambús 1 | 696 | 1083 |
| EIA | Can Roqueta/Can Revella | E119 | 1098 |
| EIA | Can Roqueta/Can Revella | E197 | 1107 |
| EIA | Can Gambús 1 | 700 | 1124 |
| EIA | Can Roqueta/Can Revella | E193 | 1133 |
| EIA | Can Roqueta/Can Revella | E284 | 1135 |
| EIA | Can Roqueta/Torre-Romeu | E258 | 1144 |
| EIA | Turó de la Font de la Canya | SJ-171 | 1147 |
| EIA | Can Roqueta-DIASA | 126 | 1170 |
| EIA | Mas d’en Boixos-1 | E-152 | 1210 |
| EIA | Mas d’en Boixos-1 | E-158 | 1214 |
| EIA | Can Gambús 1 | 707 | 1233 |
| EIA | Camp Gran | UE 4032 | 1236 |
| EIA | Mas Xirgu Sud | FS68 | 1243 |
| EIA | Can Gambús 1 | 454 | 1251 |
| EIA | La Pedrera | Sitja | 1278 |
| EIA | Can Gambús 1 | 701 | 1308 |
| EIA | Can Roqueta/Can Revella | E220 | 1316 |
| EIA | Can Roqueta/Can Revella | E54 | 1318 |
| EIA | Hort d’en Grimau | E10 | 1331 |
| EIA | Can Roqueta II (est) | 157 | 1331 |
| EIA | Can Roqueta/Can Revella | E201 | 1356 |
| EIA | Camp Gran | UE 3031 | 1360 |
| EIA | Can Roqueta/Can Revella | E46 | 1417 |
| EIA | Can Roqueta II (est) | 381 | 1465 |
| EIA | Can Gambús 1 | 717 | 1490 |
| EIA | Can Roqueta/Can Revella | E79 | 1503 |
| EIA | Can Roqueta II (est) | 768 | 1519 |
| EIA | Can Roqueta/Can Revella | E43 | 1531 |
| EIA | Can Gambús 2 | 3009 | 1536 |
| EIA | Bòbila Madurell-Can Feu | D44 | 1539 |
| EIA | Mas d’en Boixos-1 | 291 | 1544 |
| EIA | Bòbila Madurell-Can Feu | D58 | 1556 |
| EIA | Vil·la romana de Sant Amanç | UE 2 | 1584 |
| EIA | Can Roqueta-DIASA | 88 | 1600 |
| EIA | Can Roqueta II (est) | 697 | 1606 |
| EIA | Can Roqueta II (est) | 765 | 1662 |
| EIA | Can Roqueta II (est) | 767 | 1664 |
| EIA | Can Roqueta II (est) | 718 | 1723 |
| EIA | Can Roqueta II (est) | 100 | 1739 |
| EIA | Can Roqueta | E-6 | 1756 |
| EIA | Can Gambús 2 | 309 | 1780 |
| EIA | Can Roqueta-DIASA | 89 | 1781 |
| EIA | Can Roqueta/Can Revella | E78 | 1781 |
| EIA | Mas d’en Boixos-1 | E-157 | 1785 |
| EIA | Can Roqueta/Can Revella | E217 | 1794 |
| EIA | Can Roqueta-DIASA | 75 | 1822 |
| EIA | Can Roqueta/Can Revella | E100 | 1832 |
| EIA | Can Roqueta-DIASA | 97 | 1856 |
| EIA | Bòbila Madurell-Can Feu | E14 | 1898 |
| EIA | Can Roqueta II (est) | 185 | 1912 |
| EIA | Can Roqueta II (est) | 699 | 1935 |
| EIA | Bòbila Madurell-Can Feu | D26 | 1965 |
| EIA | Can Roqueta/Can Revella | E99 | 2042 |
| EIA | Can Gambús 2 | 335 | 2111 |
| EIA | Can Gambús 1 | 695 | 2127 |
| EIA | Can Roqueta/Can Revella | E224 | 2172 |
| EIA | Can Gambús 1 | 706 | 2193 |
| EIA | Can Roqueta II (est) | 181 | 2254 |
| EIA | Camp Gran | UE 4008 | 2275 |
| EIA | Can Roqueta II (est) | 708 | 2365 |
| EIA | Can Roqueta/Can Revella | E39 | 2407 |
| EIA | Can Roqueta/Can Revella | E122 | 2458 |
| EIA | Can Gambús 1 | 325 | 2469 |
| EIA | Can Roqueta II (est) | 674 | 2471 |
| EIA | Can Roqueta II (est) | 145 | 2534 |
| EIA | Can Gambús 1 | 576 | 2556 |
| EIA | Can Roqueta/Can Revella | CRCRV274 | 2559 |
| EIA | Can Roqueta-DIASA | 80 | 2573 |
| EIA | Can Roqueta II (est) | 268 | 2574 |
| EIA | Can Roqueta II (est) | 223 | 5884 |
| EIA | Can Gambús 1 | 702 | 2636 |
| EIA | Can Roqueta/Can Revella | E127 | 2646 |
| EIA | Mas d’en Boixos-1 | 448 | 2652 |
| EIA | Can Gambús 2 | 311 | 2653 |
| EIA | Can Roqueta-DIASA | 82 | 2672 |
| EIA | Mas d’en Boixos-1 | E-155 | 2697 |
| EIA | Can Roqueta II (est) | 97 | 2720 |
| EIA | El Pujolet de Moja | E29 | 2736 |
| EIA | Can Piteu I | CP-133 | 2745 |
| EIA | Can Roqueta/Can Revella | E89 | 2873 |
| EIA | Bòbila Madurell-Can Feu | D46 | 2933 |
| EIA | Can Roqueta/Can Revella | E23 | 2945 |
| EIA | Cadira del Bisbe | Sitja K | 2959 |
| EIA | Can Roqueta II (est) | 764 | 2995 |
| EIA | Can Roqueta II (est) | 107 | 3098 |
| EIA | Can Roqueta/Can Revella | E45 | 3129 |
| EIA | Can Roqueta-DIASA | 101 | 3165 |
| EIA | Can Gambús 2 | 307 | 3220 |
| EIA | Can Roqueta II (est) | 148 | 3257 |
| EIA | Can Roqueta/Can Revella | E55 | 3279 |
| EIA | Can Roqueta/Can Revella | E183 | 3307 |
| EIA | Can Roqueta/Can Revella | E211 | 3352 |
| EIA | Bòbila Madurell-Can Feu | D29 | 3371 |
| EIA | Can Gambús 2 | 319 | 3501 |
| EIA | Can Roqueta-DIASA | 85 | 3507 |
| EIA | Can Roqueta/Can Revella | E185 | 3522 |
| EIA | Can Roqueta/Can Revella | E70 | 3524 |
| EIA | Can Gambús 2 | 3042 | 3538 |
| EIA | Can Roqueta/Can Revella | E38 | 3600 |
| EIA | Can Roqueta/Can Revella | E47 | 3605 |
| EIA | Can Gambús 2 | 313 | 3607 |
| EIA | Can Roqueta/Can Revella | E182 | 3690 |
| EIA | Can Roqueta/Can Revella | E133 | 3693 |
| EIA | Can Roqueta/Can Revella | E68 | 3719 |
| EIA | Can Roqueta/Can Revella | E98 | 3723 |
| EIA | Can Roqueta II (est) | 96 | 3828 |
| EIA | Can Roqueta/Can Revella | E88 | 3872 |
| EIA | Bòbila Madurell-Can Feu | D23 | 3891 |
| EIA | Can Gambús 2 | 396 | 3900 |
| EIA | Can Gambús 1 | 699 | 3913 |
| EIA | Can Roqueta | E-5 | 4003 |
| EIA | Camp Gran | UE 3014 | 4185 |
| EIA | Can Roqueta II (est) | 191 | 4310 |
| EIA | Can Roqueta II (est) | 811 | 4479 |
| EIA | Can Roqueta/Can Revella | E95 | 4644 |
| EIA | Can Roqueta/Can Revella | E52 | 5008 |
| EIA | Turó de la Font de la Canya | SJ-154 | 7099 |
| EI | L’Era del Castell | 1629 | 217 |
| EI | Turó de la Font de la Canya | SJ-75 | 911 |
| EI | Can Roqueta II (est) | 721 | 1039 |
| EI | Turó de la Font de la Canya | SJ-43 | 1154 |
| EI | Mas Castellar | SJ-150 | 1315 |
| EI | Mas d’en Boixos-1 | 325 | 1348 |
| EI | Saus II/Camp d’en Dalmau | Sitja 44 | 1355 |
| EI | Can Roqueta II (est) | 690 | 1368 |
| EI | Saus II/Camp d’en Dalmau | Sitja 41 | 1375 |
| EI | Can Roqueta II (est) | 372 | 1375 |
| EI | Mas d’en Boixos-1 | 324 | 1414 |
| EI | Saus II/Camp d’en Dalmau | Sitja 16 | 1437 |
| EI | Can Roqueta II (est) | 146 | 1510 |
| EI | Mas Castellar | 14 | 1695 |
| EI | Can Roqueta II (est) | 696 | 1727 |
| EI | Facultat de Medicina de la UAB | E-12 | 1793 |
| EI | Mas d’en Boixos-1 | 373 | 1841 |
| EI | Mas d’en Boixos-1 | 321 | 1930 |
| EI | Mas d’en Boixos-1 | 375 | 1943 |
| EI | Can Roqueta II (est) | 704 | 1972 |
| EI | Turó de la Font de la Canya | SJ-27 | 2032 |
| EI | Can Roqueta II (est) | 153 | 2148 |
| EI | Mas Castellar | 21 | 2166 |
| EI | Mas Castellar | SJ-149 | 2361 |
| EI | Turó de la Font de la Canya | SJ-53 | 2367 |
| EI | Saus II/Camp d’en Dalmau | Sitja 7 | 2572 |
| EI | Can Gambús 3 | E-3139 | 2671 |
| EI | Saus II/Camp d’en Dalmau | Sitja 21 | 2839 |
| EI | Turó de la Font de la Canya | SJ-46 | 3029 |
| EI | Mas Castellar | SJ-155 | 3268 |
| EI | Turó de la Font de la Canya | SJ-48 | 3630 |
| EI | Turó de la Font de la Canya | SJ-12 | 3797 |
| EI | Turó de la Font de la Canya | SJ-85 | 3841 |
| EI | Mas d’en Boixos-1 | 328 | 3954 |
| EI | Turó de la Font de la Canya | SJ-59 | 3967 |
| EI | Turó de la Font de la Canya | SJ-5 | 4082 |
| EI | Turó de la Font de la Canya | SJ-88 | 4271 |
| EI | Mas Castellar | SJ-137 | 4668 |
| EI | Can Roqueta II (est) | 726 | 4689 |
| EI | Turó de la Font de la Canya | SJ-69 | 6021 |
| EI | Mas Castellar | 2 | 6592 |
| MI | Les Guàrdies | SJ-20/19 | 250 |
| MI | Mas Castellar | SJ-136 | 302 |
| MI | Puig de Sant Andreu | SJ-12 | 304 |
| MI | Sant Esteve d’Olius | SJ-218 | 321 |
| MI | Sant Esteve d’Olius | 679 | 326 |
| MI | Sant Esteve d’Olius | SJ-228 | 354 |
| MI | Bosc del Congost | Sitja 7 | 368 |
| MI | Casc Antic | UE 467 | 382 |
| MI | Camp d’en Gou/Gorg d’en Batlle | FS/SJ-88 (4045) | 443 |
| MI | Llirians del Mas, Les Torres i Sector Rec | E-45 | 475 |
| MI | Mas Castellar | SJ-133 | 487 |
| MI | Mas Castellar | SJ-116 | 492 |
| MI | Can Gambús 3 | E-2153 | 513 |
| MI | Turó de Ca n’Oliver/Ca n’Olivé | Sitja 1 | 527 |
| MI | Les Guàrdies | SJ-27/25 | 534 |
| MI | Turó del Vent | 648 | 538 |
| MI | Puig de Sant Andreu | FS-730 | 550 |
| MI | Sant Esteve d’Olius | 473 | 551 |
| MI | Mas Castellar | 12 | 565 |
| MI | Mas Castellar | 9 | 589 |
| MI | Sitges de la Carretera d’Aiguaviva | Sitja 6 (UE 2014) | 606 |
| MI | Saus II/Camp d’en Dalmau | Sitja 38 | 606 |
| MI | Turó del Vent | 611 | 627 |
| MI | Sant Esteve d’Olius | 366 | 628 |
| MI | Vial del port | Sitja 2 | 646 |
| MI | Llirians del Mas, Les Torres i Sector Rec | E-49 | 699 |
| MI | Castellvell | 04 | 703 |
| MI | Castellvell | 17 | 718 |
| MI | Llirians del Mas, Les Torres i Sector Rec | E-44 | 738 |
| MI | Saus II/Camp d’en Dalmau | Sitja 20 | 759 |
| MI | Sant Esteve d’Olius | SJ-232 | 763 |
| MI | Camp d’en Pitu Porusia | FS-17 (1005) | 765 |
| MI | Llirians del Mas, Les Torres i Sector Rec | E-12 | 769 |
| MI | Sant Esteve d’Olius | 749 | 774 |
| MI | Sant Esteve d’Olius | 685 | 794 |
| MI | Can Gambús 3 | E-2027 | 800 |
| MI | Sitges | Sitja 5 - HS | 821 |
| MI | Sitges Carrer Elisenda | E3 | 823 |
| MI | Turó del Vent | 674 | 824 |
| MI | Turó del Vent | 612 | 852 |
| MI | Bosc del Congost | Sitja 68 | 864 |
| MI | Turó del Vent | 645 | 875 |
| MI | Carrer de la Rectoria, 16 | UE 27 | 883 |
| MI | Sant Esteve d’Olius | SJ-422 | 883 |
| MI | Mas Castellar | FS-373 | 890 |
| MI | Sant Esteve d’Olius | 400 | 894 |
| MI | Facultat de Medicina de la UAB | E-11 | 894 |
| MI | Mas Castellar | SJ-119 | 904 |
| MI | Les Guàrdies | SJ-11 | 917 |
| MI | Montjuïc | 32 - 1018 | 923 |
| MI | Sant Esteve d’Olius | SJ-252 | 940 |
| MI | Bosc del Congost | Sitja 109 | 942 |
| MI | Turó de la Font de la Canya | SJ-82 | 949 |
| MI | Can Bonells | Sitja 4 | 954 |
| MI | Turó de la Font de la Canya | SJ-115 | 955 |
| MI | Turó del Vent | 684 | 973 |
| MI | Les Guàrdies | SJ-14 | 1000 |
| MI | Mas Vedruna | E-1 | 1012 |
| MI | Turó de Ca n’Oliver/Ca n’Olivé | ST-728 | 1017 |
| MI | Turó del Vent | 639 | 1017 |
| MI | Camp de l’Ylla | Sitja 2 | 1020 |
| MI | Can Gambús 2 | 107 | 1063 |
| MI | Llirians del Mas, Les Torres i Sector Rec | E-50 | 1079 |
| MI | Bosc del Congost | Sitja 42 | 1099 |
| MI | Sant Esteve d’Olius | 364 | 1130 |
| MI | Sant Esteve d’Olius | 372 | 1168 |
| MI | Turó del Vent | 614 | 1172 |
| MI | Turó del Vent | 623 | 1188 |
| MI | Olivet d’en Pujol | Sitja 2 | 1194 |
| MI | Sant Esteve d’Olius | SJ-408 | 1194 |
| MI | Sant Esteve d’Olius | SJ-150 | 1224 |
| MI | Sant Esteve d’Olius | 350 | 1224 |
| MI | Turó del Vent | 632 | 1247 |
| MI | Turó del Vent | 640 | 1247 |
| MI | Les Guàrdies | SJ-13 | 1252 |
| MI | Turó de la Font de la Canya | SJ-61 | 1267 |
| MI | Camp d’en Pitu Porusia | FS-9 (1018) | 1267 |
| MI | Sant Esteve d’Olius | 507 | 1293 |
| MI | Mas Castellar | 15 | 1320 |
| MI | Sant Sebastià de la Guarda | 2049 Sitja 3? | 1322 |
| MI | Turó del Vent | 609 | 1329 |
| MI | Mas Castellar | FS-434 | 1335 |
| MI | Sant Esteve d’Olius | SJ-167 | 1394 |
| MI | Sant Esteve d’Olius | 517 | 1406 |
| MI | Camp de l’Ylla | Sitja 1 | 1413 |
| MI | Vial del port | Sitja 4 | 1413 |
| MI | Mas Castellar | SJ-130 | 1416 |
| MI | Sant Esteve d’Olius | SJ-424 | 1416 |
| MI | Sant Esteve d’Olius | 505 | 1428 |
| MI | Sant Esteve d’Olius | 515 | 1437 |
| MI | Bosc del Congost | Sitja 41 | 1439 |
| MI | Turó del Vent | 613 | 1446 |
| MI | Sant Esteve d’Olius | 530 | 1466 |
| MI | Montjuïc | Sitja 8 - 2020 | 1485 |
| MI | Sant Esteve d’Olius | 395 | 1491 |
| MI | Saus II/Camp d’en Dalmau | Sitja 1 | 1491 |
| MI | Can Gambús 2 | 7042 | 1538 |
| MI | Far de Sant Sebastià | Sitja 10 | 1554 |
| MI | Camp d’en Gou/Gorg d’en Batlle | SJ-67 | 1558 |
| MI | Saus II/Camp d’en Dalmau | Sitja 1 | 1602 |
| MI | El Vilar | 2114 | 1614 |
| MI | Saus II/Camp d’en Dalmau | Sitja 37 | 1616 |
| MI | Llirians del Mas, Les Torres i Sector Rec | E-24 | 1622 |
| MI | Sant Esteve d’Olius | 501 | 1631 |
| MI | Sant Esteve d’Olius | 519 | 1641 |
| MI | Turó del Vent | 635 | 1643 |
| MI | Bosc del Congost | Sitja 43 | 1648 |
| MI | Camp d’en Gou/Gorg d’en Batlle | SJ-79 (4037) | 1661 |
| MI | Sant Esteve d’Olius | 753 | 1671 |
| MI | Llirians del Mas, Les Torres i Sector Rec | E-21 | 1683 |
| MI | Llirians del Mas, Les Torres i Sector Rec | E-9 | 1706 |
| MI | Sant Esteve d’Olius | 388 | 1755 |
| MI | Mas Castellar | 3A | 1764 |
| MI | Sant Esteve d’Olius | SJ-439 | 1766 |
| MI | Bosc del Congost | Sitja 50 | 1775 |
| MI | Parc del Castell de Rubí | Sitja 4 | 1811 |
| MI | Sant Esteve d’Olius | 481 | 1822 |
| MI | Sant Esteve d’Olius | 687 | 1828 |
| MI | Bosc del Congost | Sitja 36 | 1835 |
| MI | Saus II/Camp d’en Dalmau | Sitja 11 | 1839 |
| MI | Sitges de la Carretera d’Aiguaviva | Sitja 9 (UE 2021) | 1842 |
| MI | Can Sant Joan | 25 | 1855 |
| MI | Sant Esteve d’Olius | 312 | 1872 |
| MI | Les Guàrdies | SJ-26/24 | 1879 |
| MI | Turó del Vent | 630 | 1884 |
| MI | Sitges de la Carretera d’Aiguaviva | Sitja 4 (UE 2005) | 1890 |
| MI | Facultat de Medicina de la UAB | E-4 | 1915 |
| MI | Mas Castellar | FS-434 | 1922 |
| MI | Sant Esteve d’Olius | 537 | 1960 |
| MI | Vial del port | Sitja 5 | 2000 |
| MI | Sant Esteve d’Olius | SJ-244 | 2031 |
| MI | Sitges de la Carretera d’Aiguaviva | Sitja 3 (UE 2005) | 2034 |
| MI | Saus II/Camp d’en Dalmau | Sitja 17 | 2081 |
| MI | Sant Esteve d’Olius | 733 | 2085 |
| MI | LAV estructura de l’estació de la Sagrera | Sitja | 2093 |
| MI | Can Sant Joan | 111 | 2101 |
| MI | Can Sant Joan | 109 | 2102 |
| MI | Sant Esteve d’Olius | 521 | 2127 |
| MI | Turó del Vent | 637 | 2153 |
| MI | Montjuïc | 33 - 1029 | 2154 |
| MI | Sant Esteve d’Olius | 737 | 2213 |
| MI | Sant Esteve d’Olius | 528 | 2215 |
| MI | Can Gambús 3 | E-3154 | 2234 |
| MI | Estinclells | 569 | 2237 |
| MI | Bosc del Congost | Sitja 57 | 2249 |
| MI | Mas Castellar | FS-362 | 2261 |
| MI | Mas Castellar | 24 | 2291 |
| MI | Vial del port | Sitja 3 | 2302 |
| MI | Olivet d’en Pujol | Sitja 1 | 2314 |
| MI | Turó del Vent | 615 | 2336 |
| MI | Sitges | Sitja 6 - HS | 2344 |
| MI | Saus II/Camp d’en Dalmau | Sitja 40 | 2370 |
| MI | Turó del Vent | 633 | 2387 |
| MI | Mas Castellar | SJ-26 | 2394 |
| MI | Sant Esteve d’Olius | SJ-194 | 2412 |
| MI | Sant Esteve d’Olius | SJ-453 | 2415 |
| MI | Mas Castellar | SJ-30 | 2441 |
| MI | Can Gambús 1 | 348 | 2442 |
| MI | Turó de la Font de la Canya | SJ-79 | 2452 |
| MI | Cadira del Bisbe | Sitja F | 2452 |
| MI | Saus II/Camp d’en Dalmau | Sitja 2 | 2461 |
| MI | Mas Boscosa | UE 2002 | 2473 |
| MI | Mas Castellar | 18 | 2475 |
| MI | Sant Esteve d’Olius | 479 | 2528 |
| MI | Can Fatjó | UE 2 | 2540 |
| MI | Plaça de Sant Andreu | Sitja | 2560 |
| MI | Mas Castellar | 22 | 2560 |
| MI | Mas d’en Gual | 8 | 2561 |
| MI | Saus II/Camp d’en Dalmau | Sitja 19 | 2584 |
| MI | Mas Castellar | SJ-138 | 2604 |
| MI | Can Gambús 1 | 344 | 2611 |
| MI | Sant Esteve d’Olius | 535 | 2620 |
| MI | Mas Castellar | 25 | 2635 |
| MI | Turó de la Font de la Canya | SJ-31 | 2733 |
| MI | El Castellot | UE 453 | 2745 |
| MI | Can Bonells | Sitja 3 | 2767 |
| MI | Turó de la Font de la Canya | SJ-52 | 2768 |
| MI | Can Serra | 10 | 2772 |
| MI | Sant Esteve d’Olius | 503 | 2784 |
| MI | Can Gambús 3 | E-3197 | 2789 |
| MI | El Castellot | UE 216 | 2813 |
| MI | Sant Esteve d’Olius | 667 | 2822 |
| MI | Sant Esteve d’Olius | SJ-165 | 2826 |
| MI | Bosc del Congost | Sitja 32 | 2883 |
| MI | La Pleta | SJ-1111 | 2901 |
| MI | Sant Miquel de Sorba | Sitja 3 | 2918 |
| MI | Sant Esteve d’Olius | SJ-451 | 2934 |
| MI | Saus II/Camp d’en Dalmau | Sitja 43 | 2942 |
| MI | Can Xercavins | ST-265 | 2966 |
| MI | Turó de la Font de la Canya | SJ-62 | 3001 |
| MI | Sant Esteve d’Olius | 475 | 3011 |
| MI | Turó del Vent | 647 | 3044 |
| MI | Cadira del Bisbe | Sitja I (UE 1107) | 3119 |
| MI | Turó de la Font de la Canya | SJ-158 | 3174 |
| MI | El Castellot | UE 476 | 3179 |
| MI | Turó del Vent | 643 | 3203 |
| MI | Bosc del Congost | Sitja 116 | 3207 |
| MI | Turó de la Font de la Canya | SJ-64 | 3232 |
| MI | Castellvell | 25 | 3243 |
| MI | Mas Castellar | SJ-144 | 3270 |
| MI | Can Gambús 1 | 347 | 3419 |
| MI | Sant Esteve d’Olius | 310 | 3434 |
| MI | Sant Esteve d’Olius | SJ-368 | 3437 |
| MI | Turó del Vent | 694 | 3476 |
| MI | Can Gambús 2 | 4088 | 3503 |
| MI | Turó de Ca n’Oliver/Ca n’Olivé | ST-917 | 3531 |
| MI | Castellvell | 23 | 3583 |
| MI | Sant Esteve d’Olius | 362 | 3647 |
| MI | Castell de Rubí | UE 110 | 3663 |
| MI | Can Xercavins | ST-283 | 3679 |
| MI | Sant Esteve d’Olius | 511 | 3682 |
| MI | Can Gambús 1 | 345 | 3696 |
| MI | Sant Esteve d’Olius | 509 | 3706 |
| MI | Can Fatjó | UE 10 | 3708 |
| MI | Mas Castellar | SJ-134 | 3725 |
| MI | Sant Esteve d’Olius | SJ-404 | 3744 |
| MI | Sant Esteve d’Olius | 382 | 3768 |
| MI | Mas Castellar | SJ-114 | 3785 |
| MI | Mas Castellar | SJ-28 | 3790 |
| MI | Turó del Vent | 621 | 3794 |
| MI | Penya del Moro | Sitja 1 (UE 5) | 3845 |
| MI | Les Guàrdies | SJ-5 | 3920 |
| MI | Sant Esteve d’Olius | 669 | 3921 |
| MI | Mas Castellar | SJ-113 | 3928 |
| MI | Can Serra | 13 | 3955 |
| MI | Mas Castellar | SJ-103 | 3997 |
| MI | Bosc del Congost | Sitja 88 | 4039 |
| MI | Can Sant Joan | 110 | 4061 |
| MI | Castellvell | 09 | 4082 |
| MI | Sant Sebastià de la Guarda | 2039 Sitja 2 | 4085 |
| MI | Turó de Ca n’Oliver/Ca n’Olivé | Sitja 292 | 4112 |
| MI | Mas Castellar | SJ-15 | 4115 |
| MI | Les Guàrdies | SJ-30/28 | 4120 |
| MI | Sant Esteve d’Olius | 539 | 4138 |
| MI | Turó de la Font de la Canya | SJ-70 | 4155 |
| MI | Can Gambús 1 | 346 | 4173 |
| MI | Can Serra | 12 | 4185 |
| MI | Turó de la Font de la Canya | SJ-121 | 4289 |
| MI | Can Serra | 1 | 4308 |
| MI | El sitjar dels Escalers/Camp dels Escalers | Sitja 16 (nº3-1997) | 4322 |
| MI | Facultat de Medicina de la UAB | E-6 | 4339 |
| MI | Can Gambús 2 | 199 | 4345 |
| MI | Mas Castellar | 4C | 4378 |
| MI | Les Guàrdies | SJ-9 | 4414 |
| MI | Can Gambús 3 | E-3333 | 4475 |
| MI | Mas Castellar | SJ-1 | 4486 |
| MI | Bosc del Congost | Sitja 38 | 4497 |
| MI | Les Guàrdies | SJ-21/20 | 4623 |
| MI | Sant Sebastià de la Guarda | SJ-6 | 4705 |
| MI | Sant Esteve d’Olius | 317 | 4710 |
| MI | Sant Sebastià de la Guarda | Sitja 10 | 4806 |
| MI | Poblat ibèric de Castell | 2845 | 4818 |
| MI | Llirians del Mas, Les Torres i Sector Rec | E-20 | 4904 |
| MI | Castellvell | 06 | 4957 |
| MI | El sitjar dels Escalers/Camp dels Escalers | Sitja 15 (nº2-1997) | 4957 |
| MI | Castellvell | 12 | 4989 |
| MI | Turó de la Font de la Canya | SJ-18 | 5022 |
| MI | Can Serra | 9 | 5033 |
| MI | Turó del Vent | 608 | 5080 |
| MI | Mas Castellar | SJ-27 | 5089 |
| MI | Sant Esteve d’Olius | 386 | 5224 |
| MI | Montjuïc | Sitja 16 - 2045 = Serra 3 | 5384 |
| MI | Turó de la Font de la Canya | SJ-54 | 5441 |
| MI | Sant Esteve d’Olius | 340 | 5515 |
| MI | Sant Esteve d’Olius | 305 | 5540 |
| MI | Sant Esteve d’Olius | SJ-449 | 5562 |
| MI | Les Guàrdies | SJ-3 | 5836 |
| MI | Bosc del Congost | Sitja 40 | 5848 |
| MI | Mas Castellar | 4B | 5883 |
| MI | Turó de la Font de la Canya | SJ-184 | 6008 |
| MI | El sitjar dels Escalers/Camp dels Escalers | Sitja 2 | 6230 |
| MI | Turó de la Font de la Canya | SJ-11 | 6299 |
| MI | Les Guàrdies | SJ-1 | 6465 |
| MI | Turó de la Font de la Canya | SJ-65 | 6519 |
| MI | Turó de la Font de la Canya | SJ-9 | 6594 |
| MI | Turó del Vent | 682 | 6611 |
| MI | Mas Castellar | SJ-31 | 6635 |
| MI | Can Gambús 1 | 301 | 6760 |
| MI | Mas Castellar | 10 | 7024 |
| MI | Mas Castellar | SJ-153 | 7085 |
| MI | Mas Castellar | SJ-25 | 7179 |
| MI | Turó de la Font de la Canya | SJ-176 | 7255 |
| MI | Turó de la Font de la Canya | SJ-14 | 7502 |
| MI | Parc Residencial Vilacolum | Estructura 3 | 8872 |
| MI | Mas Castellar | 1 | 8998 |
| MI | Turó de la Font de la Canya | SJ-56 | 9068 |
| MI | Bosc del Congost | Sitja 3 | 9760 |
| MI | Turó de Ca n’Oliver/Ca n’Olivé | Sitja 293 | 9916 |
| MI | El Vilar | 2111 | 10423 |
| MI | Sant Sebastià de la Guarda | 1111 (SJ-4) | 11930 |
| MI | Can Xercavins | ST-264 | 13701 |
| MI | Montjuïc | Sitja 2 - 2003 | 45953 |
| MI | Montjuïc | Sitja 17 - 2068 = Serra 4 | 61034 |
| LI | Turó de Ca n’Oliver/Ca n’Olivé | FS-926 | 33 |
| LI | Camp de l’Arrencada | Sitja 4 | 72 |
| LI | Turó de Ca n’Oliver/Ca n’Olivé | ST-753 | 113 |
| LI | Turó del Vent | 610 | 114 |
| LI | Camp de les Lloses | UE 2221 | 126 |
| LI | Planell de Sanaüja | UE 34 | 154 |
| LI | Turó de Ca n’Oliver/Ca n’Olivé | ST-749 | 163 |
| LI | Camp del Pla de Sant Esteve | FS-68 | 231 |
| LI | Turó de Ca n’Oliver/Ca n’Olivé | ST-922 | 239 |
| LI | Les Guàrdies | SJ-28/26 | 251 |
| LI | Les Guàrdies | SJ-29/27 | 256 |
| LI | Prolongació del Carrer de la Pau | Sitja 7 | 262 |
| LI | Pla de les Sitges del Camaró | Sitja 2 | 277 |
| LI | Bosc del Congost | Sitja 60 | 282 |
| LI | Can Gambús 2 | 132 | 288 |
| LI | Camp del Pla de Sant Esteve | FS-72 | 333 |
| LI | Camp del Pla de Sant Esteve | FS-42 | 339 |
| LI | Pla de les Sitges del Camaró | Sitja 1 | 346 |
| LI | Camp del Pla de Sant Esteve | FS-66 | 392 |
| LI | Turó del Vent | 606 | 392 |
| LI | Camp Gran | UE 3123 | 410 |
| LI | Casc Antic | UE 469 | 412 |
| LI | El Castellot | 830 | 445 |
| LI | Pla de les Sitges del Camaró | Sitja 8 | 448 |
| LI | Turó de Ca n’Oliver/Ca n’Olivé | Sitja 3 | 475 |
| LI | Turó del Vent | 680 | 483 |
| LI | Camp Gran | UE 3027 | 491 |
| LI | Turó de la Rovira | Sitja 4 | 522 |
| LI | Turó de Ca n’Oliver/Ca n’Olivé | ST-927 | 538 |
| LI | Oppidum de Burriac | SJ-1 (B-CM) | 570 |
| LI | Camp Gran | UE 3000 | 576 |
| LI | Serrat dels Espinyers | 1136 | 606 |
| LI | Pla de les Sitges del Camaró | Sitja 4 | 621 |
| LI | Casc Antic | UE 697 | 625 |
| LI | C/Alou, núm. 43- 47 i C/ Mossèn Francesc Albertí, núm. 1- 3 | 627 | 636 |
| LI | Camp Gran | UE 3018 | 637 |
| LI | Can Gambús 3 | E-2217 | 643 |
| LI | Camp Gran | UE 3004 | 690 |
| LI | Casc Antic | UE 660 | 692 |
| LI | Turó de Ca n’Oliver/Ca n’Olivé | ST-729 | 701 |
| LI | Pla de les Sitges del Camaró | Sitja 7 | 708 |
| LI | Bosc del Congost | Sitja 83 | 713 |
| LI | Turó de Ca n’Oliver/Ca n’Olivé | Sitja 38 | 718 |
| LI | Carrer de la Rectoria, 16 | UE 25 | 726 |
| LI | Oppidum de Burriac | SJ-2 (B-CM) | 735 |
| LI | Camp de les Lloses | UE 2108 | 737 |
| LI | Facultat de Medicina de la UAB | E-16 | 755 |
| LI | Bosc del Congost | Sitja 93 | 759 |
| LI | Camp Gran | UE 4089 | 762 |
| LI | Camp Gran | UE 3022 | 778 |
| LI | Puig Castellar | 10 | 784 |
| LI | Oppidum de Burriac | SJ-3 (B-CM) | 788 |
| LI | Torre Vedruna | Sitja 32 | 800 |
| LI | Camp Gran | UE 4047 | 835 |
| LI | Mas d’en Gual | 17 | 846 |
| LI | Camp Gran | UE 3040 | 863 |
| LI | Torre Vedruna | Sitja 16 | 882 |
| LI | Poblat ibèric de Castellvell | 68 | 882 |
| LI | Camp Gran | UE 4022 | 889 |
| LI | Camp de l’Arrencada | Sitja 18 | 904 |
| LI | Prolongació del Carrer de la Pau | Sitja 4 | 905 |
| LI | Camp Gran | UE 3012 | 914 |
| LI | Camp Gran | UE 3002 | 921 |
| LI | Casc Antic | UE 623 | 941 |
| LI | Puig Castellar | 11 | 950 |
| LI | Camp Gran | UE 3116 | 965 |
| LI | Turó de la Bateria o Puig d’en Roca III - Vials | E-4 | 979 |
| LI | Les Guàrdies | SJ-2 | 981 |
| LI | Poblat ibèric de Castellvell | 56 | 984 |
| LI | Camp Gran | UE 3038 | 1021 |
| LI | Can Gambús 3 | E-1109 | 1022 |
| LI | Mas d’en Gual | 4 | 1032 |
| LI | Poblat ibèric de Castellvell | 58 | 1045 |
| LI | Can Gambús 2 | 877 | 1067 |
| LI | Bosc del Congost | Sitja 16 | 1071 |
| LI | Oppidum de Burriac | SJ-8 (B-CM) | 1074 |
| LI | Bosc del Congost | Sitja 8 | 1083 |
| LI | Camp del Pla de Sant Esteve | FS-53 | 1083 |
| LI | Turó del Vent | 644 | 1089 |
| LI | Bosc del Congost | Sitja 84 | 1099 |
| LI | Camp de l’Arrencada | Sitja 8 | 1102 |
| LI | Mas Boscà | Sitja | 1105 |
| LI | Can Gambús 3 | E-1062 | 1106 |
| LI | Bosc del Congost | Sitja 66 | 1111 |
| LI | Carrer de la Rectoria, 16 | UE 41 | 1112 |
| LI | Poblat ibèric de Castellvell | 44 | 1128 |
| LI | Camp Gran | UE 3114 | 1144 |
| LI | Camp Gran | UE 3054 | 1146 |
| LI | Bosc del Congost | Sitja 15 | 1168 |
| LI | Camp Gran | UE 4080 | 1190 |
| LI | Camp Gran | UE 4058 | 1190 |
| LI | Vinya del Ticó | Sitja | 1193 |
| LI | Oppidum de Burriac | SJ-10 (B-CM) | 1216 |
| LI | Can Tintorer | Sitja 2 | 1221 |
| LI | Can Miralles - Can Modolell (Oppidum Burriac) | Sitja 38 | 1241 |
| LI | Camp Gran | UE 4020 | 1242 |
| LI | Torre Vedruna-Sector Transports | E82 | 1269 |
| LI | Turó del Vent | 602 | 1316 |
| LI | Poblat ibèric de Castellvell | 48 | 1327 |
| LI | Camp Gran | UE 3006 | 1334 |
| LI | Turó de Ca n’Oliver/Ca n’Olivé | ST-921 | 1346 |
| LI | C/Alou, núm. 43- 47 i C/ Mossèn Francesc Albertí, núm. 1- 3 | 610 | 1391 |
| LI | Camp de l’Arrencada | Sitja 17 | 1392 |
| LI | Sitges | Sitja 3 - UE 108 | 1412 |
| LI | Oppidum de Burriac | SJ-5 (B-CM) | 1417 |
| LI | Bosc del Congost | Sitja 59 | 1424 |
| LI | Can Miralles - Can Modolell (Oppidum Burriac) | SJ-34 (B-CM) | 1424 |
| LI | Puig del Castell | Sitja 2 (UE 1044) | 1446 |
| LI | Mas Vilalba | MV-1 | 1469 |
| LI | Camp Gran | UE 4071 | 1476 |
| LI | Poblat ibèric de Castellvell | 36 | 1488 |
| LI | Empúries | Gall 1 | 1507 |
| LI | Camp de les Lloses | UE 2227 | 1514 |
| LI | C/Alou, núm. 43- 47 i C/ Mossèn Francesc Albertí, núm. 1- 3 | 628 | 1523 |
| LI | El Castellot | 827 | 1526 |
| LI | Camp del Pla de Sant Esteve | FS-63 | 1538 |
| LI | Bosc del Congost | Sitja 4 | 1583 |
| LI | Turó de Ca n’Oliver/Ca n’Olivé | ST-776 | 1584 |
| LI | Can Tintorer | Sitja 1 | 1589 |
| LI | Bosc del Congost | Sitja 45 | 1591 |
| LI | Oppidum de Burriac (poblat + sitges) | SJ-4 (B-CM) | 1594 |
| LI | Facultat de Medicina de la UAB | E-9 | 1595 |
| LI | Serrat dels Espinyers | 1063 | 1618 |
| LI | Can Pona | 2 | 1621 |
| LI | C/Alou, núm. 43- 47 i C/ Mossèn Francesc Albertí, núm. 1- 3 | 618 | 1648 |
| LI | Can Gambús 2 | 805 | 1657 |
| LI | Can Gambús 3 | E-3033 | 1662 |
| LI | Planell de Sanaüja | UE 8 | 1667 |
| LI | Torre Vedruna | Sitja 29 | 1683 |
| LI | Camp del Pla de Sant Esteve | FS-24 | 1695 |
| LI | Can Gambús 3 | E-2067 | 1705 |
| LI | Prolongació del Carrer de la Pau | Sitja 1 | 1716 |
| LI | Sector industrial el rentador. Sector sud | Sitja 14 (UE 3) | 1721 |
| LI | Camp de l’Arrencada | Sitja 11 | 1725 |
| LI | El Vilar | 2308=2213 | 1727 |
| LI | Bosc del Congost | Sitja 46 | 1740 |
| LI | Bosc del Congost | Sitja 115 | 1741 |
| LI | Can Gambús 2 | 832 | 1747 |
| LI | Camí de Can Segarra-Can Bartomeu (Oppidum Burriac) | SJ-33 | 1780 |
| LI | Montjuïc | Sitja C (UE 1049) | 1795 |
| LI | Bosc del Congost | Sitja 98 | 1808 |
| LI | Can Gambús 3 | E-2038 | 1835 |
| LI | Bosc del Congost | Sitja 48 | 1837 |
| LI | Serrat dels Espinyers | 1033 | 1839 |
| LI | Can Gambús 2 | 641 | 1853 |
| LI | Bosc del Congost | Sitja 120 | 1856 |
| LI | Sector industrial el rentador. Sector sud | Sitja 15 (UE 5) | 1863 |
| LI | Can Gambús 2 | 105 | 1864 |
| LI | Can Gambús 3 | E-3223 | 1888 |
| LI | Turó de Ca n’Oliver/Ca n’Olivé | ST-783 | 1914 |
| LI | Oppidum de Burriac | SJ-28 (B-CM) | 1923 |
| LI | Can Gambús 2 | 853 | 1953 |
| LI | Serrat dels Espinyers | 1090 | 1980 |
| LI | Sector industrial el rentador. Sector sud | Sitja 20 (UE 15) | 1983 |
| LI | Camp de l’Arrencada | Sitja 5 | 1989 |
| LI | Bosc del Congost | Sitja 21 | 1990 |
| LI | Sant Sebastià de la Guarda | 3037 | 2003 |
| LI | Bosc del Congost | Sitja 65 | 2017 |
| LI | Camp del Pla de Sant Esteve | FS-31 | 2035 |
| LI | Serrat dels Espinyers | 1031 | 2077 |
| LI | Can Gambús 2 | 192 | 2086 |
| LI | Facultat de Medicina de la UAB | E-1 | 2115 |
| LI | Can Gambús 3 | E-1003 | 2119 |
| LI | Carrer de l’Hostal del Pi, Polígon Barcelonès | UE 5 | 2120 |
| LI | Puig Castellar | 1 | 2120 |
| LI | Can Miralles - Can Modolell (Oppidum Burriac) | SJ-31 (B-CM) | 2145 |
| LI | Bosc del Congost | Sitja 31 | 2158 |
| LI | Serrat dels Espinyers | 1193 | 2207 |
| LI | El Castellot | UE 189 | 2208 |
| LI | Turó de Ca n’Oliver/Ca n’Olivé | Sitja 35 | 2220 |
| LI | Puig Castellar | 8 | 2254 |
| LI | Sector industrial el rentador. Sector sud | Sitja 17 (UE 13) | 2259 |
| LI | Sant Sebastià de la Guarda | SJ-8 | 2270 |
| LI | El Molàs | 3 | 2295 |
| LI | El Molàs | 4 | 2295 |
| LI | Bosc del Congost | Sitja 19 | 2309 |
| LI | Bosc del Congost | Sitja 13 | 2312 |
| LI | Can Gambús 3 | E-2023 | 2324 |
| LI | Turó de Ca n’Oliver/Ca n’Olivé | ST-784 | 2346 |
| LI | Turó de Ca n’Oliver/Ca n’Olivé | Sitja 39 | 2355 |
| LI | El Castellot | UE 9 | 2359 |
| LI | Can Gambús 2 | 867 | 2373 |
| LI | Bosc del Congost | Sitja 106 | 2419 |
| LI | Serrat dels Espinyers | 1109 | 2431 |
| LI | Facultat de Medicina de la UAB | E-14 | 2453 |
| LI | C/Alou, núm. 43- 47 i C/ Mossèn Francesc Albertí, núm. 1- 3 | 621 | 2461 |
| LI | Bosc del Congost | Sitja 34 | 2465 |
| LI | Bosc del Congost | Sitja 47 | 2475 |
| LI | Can Gambús 3 | E-1005 | 2481 |
| LI | Puig Castellar | 6 | 2486 |
| LI | Bosc del Congost | Sitja 56 | 2490 |
| LI | Sector industrial el rentador. Sector sud | Sitja 19 (UE 9) | 2522 |
| LI | Camp Gran | UE 3127 | 2537 |
| LI | Saus II/Camp d’en Dalmau | Sitja 33 | 2565 |
| LI | Turó de Ca n’Oliver/Ca n’Olivé | Sitja 52 | 2570 |
| LI | Bosc del Congost | Sitja 29 | 2571 |
| LI | Turó de Ca n’Oliver/Ca n’Olivé | ST-785 | 2571 |
| LI | Can Gambús 3 | E-1105 | 2585 |
| LI | Turó de Ca n’Oliver/Ca n’Olivé | ST-782 | 2636 |
| LI | Torre Vedruna | Sitja 28 | 2640 |
| LI | Bosc del Congost | Sitja 58 | 2644 |
| LI | El Vilar | 2109 | 2645 |
| LI | Serrat dels Espinyers | 1231 | 2648 |
| LI | Mas Castellar | SJ-156 | 2649 |
| LI | Camí de Can Segarra-Can Bartomeu (Oppidum Burriac) | SJ-1 | 2669 |
| LI | Torre Vedruna-Sector Transports | E91 | 2681 |
| LI | Sector industrial el rentador. Sector sud | Sitja 16 (UE 11) | 2693 |
| LI | Puig Castellar | 9 | 2699 |
| LI | Torre Vedruna-Sector Transports | E93 | 2701 |
| LI | Camp del Pla de Sant Esteve | FS-8 | 2708 |
| LI | Bosc del Congost | Sitja 35 | 2731 |
| LI | Bosc del Congost | Sitja 18 | 2772 |
| LI | Camp del Pla de Sant Esteve | FS-20 | 2772 |
| LI | Sitges | Sitja 4 - HA | 2786 |
| LI | Torre Vedruna-Sector Transports | E106 | 2791 |
| LI | Plaça de la Constitució | UE 7 | 2793 |
| LI | Turó de Ca n’Oliver/Ca n’Olivé | ST-778 | 2844 |
| LI | Torre Vedruna-Sector Transports | E105 | 2877 |
| LI | Can Gambús 2 | 857 | 2922 |
| LI | Les Guàrdies | SJ-25/23 | 2945 |
| LI | Ca l’Almell de la Muntanya | Sitja 1 | 2948 |
| LI | Can Miralles - Can Modolell (Oppidum Burriac) | SJ-30 (B-CM) | 2984 |
| LI | Missatges | 6 | 2994 |
| LI | Can Gambús 3 | E-2064 | 3013 |
| LI | Bosc del Congost | Sitja 89 | 3013 |
| LI | Santa Creu d’Olorda | Sitja 1 | 3037 |
| LI | Barranc del Prat | UE 107 | 3063 |
| LI | Can Gambús 2 | 156 | 3075 |
| LI | Turó de la Rovira | Sitja 2 | 3076 |
| LI | Puig Castellar | 7 | 3076 |
| LI | Torre Vedruna | Sitja 33 | 3089 |
| LI | Camp del Pla de Sant Esteve | FS-25 | 3130 |
| LI | Can Gambús 3 | E-1007 | 3143 |
| LI | Facultat de Medicina de la UAB | E-13 | 3146 |
| LI | Poblat ibèric de Castell | 2813 | 3147 |
| LI | Serrat dels Espinyers | 1165 | 3176 |
| LI | Mas Castellar | SJ-101 | 3176 |
| LI | Camp del Pla de Sant Esteve | FS-37 | 3177 |
| LI | Camp del Pla de Sant Esteve | FS-27 | 3191 |
| LI | Serrat dels Espinyers | 1197 | 3214 |
| LI | Serrat dels Espinyers | 1233 | 3230 |
| LI | Sant Sebastià de la Guarda | Sitja 14 (1622) | 3268 |
| LI | Turó de Ca n’Oliver/Ca n’Olivé | Sitja 50 | 3294 |
| LI | Missatges | 5 | 3300 |
| LI | Torre Vedruna-Sector Transports | E83 | 3321 |
| LI | Camp del Pla de Sant Esteve | FS-36 | 3329 |
| LI | Poblat ibèric de Castell | 3234 | 3352 |
| LI | El Castellot | UE 24 | 3383 |
| LI | Torre Vedruna | Sitja 30 | 3399 |
| LI | Turó de Ca n’Oliver/Ca n’Olivé | Sitja 51 | 3413 |
| LI | Camp del Pla de Sant Esteve | FS-40 | 3455 |
| LI | Turó de Ca n’Oliver/Ca n’Olivé | ST-781 | 3461 |
| LI | Turó de la Rovira | Sitja 3 | 3461 |
| LI | Torre Vedruna-Sector Transports | E109 | 3481 |
| LI | Can Gambús 3 | E-1101 | 3498 |
| LI | El Molàs | 2 | 3531 |
| LI | Camp del Pla de Sant Esteve | FS-39 | 3539 |
| LI | Serrat dels Espinyers | 1290 | 3567 |
| LI | Camp de l’Abadia | S-24 | 3573 |
| LI | Bosc del Congost | Sitja 53 | 3575 |
| LI | Camp del Pla de Sant Esteve | FS-43 | 3575 |
| LI | Puig Castellar | 3 | 3576 |
| LI | Camp de l’Abadia | S-2 | 3616 |
| LI | Rosella | 138 | 3631 |
| LI | El Castellot | UE 25 | 3650 |
| LI | Turó de Ca n’Oliver/Ca n’Olivé | ST-780 | 3667 |
| LI | Bosc del Congost | Sitja 5 | 3676 |
| LI | Sitja de ca l’Angusto | Sitja | 3736 |
| LI | Serrat dels Espinyers | 1297 | 3800 |
| LI | Camp del Pla de Sant Esteve | FS-14 | 3852 |
| LI | Torre Vedruna-Sector Transports | E43 | 3858 |
| LI | Font d’Abril/Font de Bril | Sitja | 3885 |
| LI | Mas d’en Gual | 2 | 3885 |
| LI | Turó de la Font de la Canya | SJ-191 | 3886 |
| LI | Can Gambús 3 | E-2029 | 3920 |
| LI | Camp de l’Abadia | S-30 | 3939 |
| LI | Oppidum de Burriac | SJ-6 (B-CM) | 3952 |
| LI | Turó de Ca n’Oliver/Ca n’Olivé | ST-777 | 3965 |
| LI | Bosc del Congost | Sitja 72 | 3966 |
| LI | Camp de l’Abadia | S-17 | 3999 |
| LI | Turó de Ca n’Oliver/Ca n’Olivé | ST-787 | 3999 |
| LI | Puig Castellar | 4 | 4061 |
| LI | Serrat dels Espinyers | 1179 | 4061 |
| LI | Castellvell | 5 | 4080 |
| LI | Turó de la Rovira | Sitja 13 | 4153 |
| LI | El Vilar | 2014 | 4174 |
| LI | Serrat dels Espinyers | 1183 | 4179 |
| LI | Carrer de la Rectoria, 16 | UE 45 | 4189 |
| LI | Serrat dels Espinyers | 1177 | 4251 |
| LI | Bosc del Congost | Sitja 2 | 4251 |
| LI | Turó de Ca n’Oliver/Ca n’Olivé | ST-779 | 4263 |
| LI | Castell de Rubí | UE 121 | 4276 |
| LI | Camp de l’Abadia | S-18 | 4326 |
| LI | Guissona (fosses del camp 1) | 1 | 4400 |
| LI | Serrat dels Espinyers | 1317 | 4414 |
| LI | Sitja de la Rambla Onze de Setembre | Sitja 1 | 4416 |
| LI | Torre Vedruna-Sector Transports | E87 | 4441 |
| LI | Torre Vedruna-Sector Transports | E90 | 4518 |
| LI | Rosella | 5 | 4522 |
| LI | El Castellot | 757 | 4539 |
| LI | Prolongació del Carrer de la Pau | Sitja 9 | 4619 |
| LI | El Castellot | UE 370 | 4622 |
| LI | Camp del Pla de Sant Esteve | FS-21 | 4714 |
| LI | Els Mallols | E-138 | 4735 |
| LI | Serrat dels Espinyers | 1004 | 4741 |
| LI | Torre Vedruna-Sector Transports | E84 | 4782 |
| LI | Rosella | 10 | 4783 |
| LI | Serrat dels Espinyers | 1315 | 4805 |
| LI | Sant Sebastià de la Guarda | 3043 | 4831 |
| LI | Facultat de Medicina de la UAB | E-8 | 4862 |
| LI | Les Guàrdies | SJ-24/22 | 4866 |
| LI | Can Miralles - Can Modolell (Oppidum Burriac) | SJ-22 (B-CM) | 4957 |
| LI | Mas d’en Gual | 3 | 4985 |
| LI | Serrat dels Espinyers | 1185 | 5024 |
| LI | Carrer de la Rectoria, 16 | UE 39 | 5060 |
| LI | Torre Vedruna-Sector Transports | E104 | 5095 |
| LI | Sant Sebastià de la Guarda | 3045 | 5103 |
| LI | Turó de Ca n’Oliver/Ca n’Olivé | Sitja 54 | 5136 |
| LI | Turó de la Rovira | Sitja 6 | 5298 |
| LI | Torre Vedruna-Sector Transports | E100 | 5313 |
| LI | Sant Sebastià de la Guarda | 3035 | 5407 |
| LI | Torre Vedruna-Sector Transports | E86 | 5468 |
| LI | Puig Castellar | 5 | 5525 |
| LI | Sant Joan Nord | UE 125 | 5536 |
| LI | Bosc del Congost | Sitja 91 | 5545 |
| LI | El Castellot | UE 206 | 5564 |
| LI | Camp de l’Abadia | S-26 | 5581 |
| LI | Els Mallols | E-153 | 5587 |
| LI | Turó de Ca n’Oliver/Ca n’Olivé | Sitja 37 | 5623 |
| LI | C/Alou, núm. 43- 47 i C/ Mossèn Francesc Albertí, núm. 1- 3 | 615 | 5652 |
| LI | Camp de l’Abadia | S-20 | 5658 |
| LI | Camp del Pla de Sant Esteve | FS-19 | 5736 |
| LI | Mas d’en Gual | 5 | 5878 |
| LI | Turó de la Rovira | Sitja 1 | 5897 |
| LI | Sector industrial el rentador. Sector sud | Sitja 4 | 5933 |
| LI | Serrat dels Espinyers | 1163 | 5965 |
| LI | Serrat dels Espinyers | 1221 | 6041 |
| LI | Torre Vedruna | Sitja 10 | 6176 |
| LI | Facultat de Medicina de la UAB | E-15 | 6176 |
| LI | Turó del Vent | 678 | 6178 |
| LI | Serrat dels Espinyers | 1278 | 6203 |
| LI | Serrat dels Espinyers | 1303 | 6233 |
| LI | Sant Sebastià de la Guarda | SJ-5 | 6234 |
| LI | Sant Sebastià de la Guarda | 2064 Sitja 4 | 6278 |
| LI | Turó de Ca n’Oliver/Ca n’Olivé | ST-774 | 6323 |
| LI | Can Badell/Can Vedell/CEIP el Turó | Sitja 3 (UE 131) | 6338 |
| LI | Camp de l’Abadia | S-12 | 6443 |
| LI | El Vilar | 2126 | 6464 |
| LI | Camp de l’Abadia | S-16 | 6495 |
| LI | Turó de Ca n’Oliver/Ca n’Olivé | ST-773 | 6532 |
| LI | Can Miralles - Can Modolell (Oppidum Burriac) | SJ-35 (B-CM) | 6565 |
| LI | Turó de Ca n’Oliver/Ca n’Olivé | ST-788 | 6629 |
| LI | Sitges | Sitja 5 - UE 125 | 6710 |
| LI | Poblat ibèric de Castell | 3235 | 6831 |
| LI | Rosella | 102 | 6852 |
| LI | Sant Joan Nord | UE 49 | 6906 |
| LI | Camp de l’Abadia | S-13 | 6972 |
| LI | Sector industrial el rentador. Sector sud | Sitja 8 | 7438 |
| LI | Can Miralles - Can Modolell (Oppidum Burriac) | SJ-33 (B-CM) | 7475 |
| LI | Turó de Ca n’Oliver/Ca n’Olivé | ST-775 | 7475 |
| LI | Sector industrial el rentador. Sector sud | Sitja 6 | 7551 |
| LI | Les Guàrdies | SJ-12 | 7553 |
| LI | Hospital de la Santa Creu i Sant Pau | Sitja 309 | 7691 |
| LI | El Castellot | UE 410 | 8061 |
| LI | Turó de Ca n’Oliver/Ca n’Olivé | Sitja 41 | 8071 |
| LI | LAV estructura de l’estació de la Sagrera | Sitja | 8130 |
| LI | Puig Castellar | 2 | 8162 |
| LI | Camp del Pla de Sant Esteve | FS-4 | 8417 |
| LI | Turó de Ca n’Oliver/Ca n’Olivé | ST-789 | 9087 |
| LI | Hospital de la Santa Creu i Sant Pau | 2a sitja | 9138 |
| LI | Turó de Ca n’Oliver/Ca n’Olivé | ST-786 | 9300 |
| LI | Turó de Ca n’Oliver/Ca n’Olivé | Sitja 36 | 10125 |
| LI | Hospital de la Santa Creu i Sant Pau | Sitja 1008 | 10299 |
| LI | Les Guàrdies | SJ-10 | 10493 |
| LI | Sant Joan Nord | UE 91 | 10609 |
| LI | Missatges | 12 | 12244 |
| LI | Puig Castellar | Sitja 1 | 15945 |
| LI | Planell de Sanaüja | UE 4 | 17540 |
| LI | Sant Sebastià de la Guarda | Sitja 12 | 22810 |
